# Novel interconnections of HOG signaling revealed by combined use of two proteomic software packages

**DOI:** 10.1186/s12964-019-0381-z

**Published:** 2019-06-17

**Authors:** Marion Janschitz, Natalie Romanov, Gina Varnavides, David Maria Hollenstein, Gabriela Gérecová, Gustav Ammerer, Markus Hartl, Wolfgang Reiter

**Affiliations:** 1 Department of Biochemistry, Max F. Perutz Laboratories, Vienna BioCenter, Vienna, Austria; 2 Children’s Cancer Research Institute, St. Anna Kinderspital, Vienna, Austria; 3 Structural and Computational Biology Unit, European Molecular Biology Laboratory, Meyerhofstrasse 1, 69117 Heidelberg, Germany; 4 Mass Spectrometry Facility, Max F. Perutz Laboratories, University of Vienna, Vienna BioCenter, Vienna, Austria; 5 Current Address: Department of Molecular Sociology, Max Planck Institute of Biophysics, 60438 Frankfurt am Main, Germany

**Keywords:** Proteome discoverer, MaxQuant, Proteomics, Mitogen-activated protein kinase (MAPK), Hyperosmotic stress response, High-osmolarity glycerol (HOG), Hog1, Kic1, Orm2, ORMDL, p38

## Abstract

Modern quantitative mass spectrometry (MS)-based proteomics enables researchers to unravel signaling networks by monitoring proteome-wide cellular responses to different stimuli. MS-based analysis of signaling systems usually requires an integration of multiple quantitative MS experiments, which remains challenging, given that the overlap between these datasets is not necessarily comprehensive. In a previous study we analyzed the impact of the yeast mitogen-activated protein kinase (MAPK) Hog1 on the hyperosmotic stress-affected phosphorylome. Using a combination of a series of hyperosmotic stress and kinase inhibition experiments, we identified a broad range of direct and indirect substrates of the MAPK. Here we re-evaluate this extensive MS dataset and demonstrate that a combined analysis based on two software packages, MaxQuant and Proteome Discoverer, increases the coverage of Hog1-target proteins by 30%. Using protein-protein proximity assays we show that the majority of new targets gained by this analysis are indeed Hog1-interactors. Additionally, kinetic profiles indicate differential trends of Hog1-dependent versus Hog1-independent phosphorylation sites. Our findings highlight a previously unrecognized interconnection between Hog1 signaling and the RAM signaling network, as well as sphingolipid homeostasis.

**Electronic supplementary material:**

The online version of this article (10.1186/s12964-019-0381-z) contains supplementary material, which is available to authorized users.

## Methods

### Yeast strain and plasmid construction

Yeast strains used in M-track assays were generated as described in Brezovich et al., [1]. WR1242 (S288c HOG1-TEV-ProteinA-Histone3-HA, Mat a) was obtained by transforming a S288c *HOG1*-GFP strain of the yeast strain-library available from Life Technologies (http://clones.lifetechnologies.com; [2]) with PacI/SacI cut plasmid pCK902, encoding the TEV-ProteinA-Histone3-HA cassette [1]. WR1249 (S288c HOG1-TEV-ProteinA-Histone3-HA, Mat α) was obtained from backcrossing WR1242 with a S288c wild type, Mat α strain. M-track strain WR1288 was obtained by transformation of a S288c *NUP2*-GFP strain (Mat a) [2] with PacI/PmeI restriction digests of plasmid pCK900, encoding the myc-HKMT tagging cassette [1]. Positively tested transformants were crossed with WR1249 resulting in the final M-track strains. M-track strains MJ314 - MJ369 were obtained by transformation of WR1242 with PCR amplifications of the myc-HKMT tagging cassette. For PCR reactions a modified version of plasmid pCK900 (pJA31 - unpublished material kindly provided by Jillian Augustine) and corresponding primers designed according to Knop et al. [3] were used. M-track strains MJ428 - MJ440 were created similarly by transforming WR1249. M-track strain GV1 was obtained by transforming PCR amplifications of a N-terminal tagging cassette from plasmid pMJ089 (*LEU2*-*TPI1* promoter-MYC-HKMT-GL (glycine linker)-*YSP2* (derivative of YIPlac211)) into WR1249. Standard genetic manipulations methods were used to create pMJ089. Strains GG612 and GG616 were obtained by transformation of WR557 [4] with PCR amplifications of HB tagging cassettes from plasmids pWR160 [5], pWR268 [5] and pFA6a-HBH-TRP1 [6]. GG617 was obtained by transformation of W303 Hog1as with a standard HA tagging cassette. All strains and Plasmids used in this study are listed in Additional file 12: Table S7.

### Growth conditions

Yeast cells were grown shaking (200 rpm) at 30 °C in synthetic medium (0.17% yeast nitrogen base, 0.5% ammonium sulfate, 2% glucose, and amino acids as required) or rich medium (YPD; 1% yeast extract, 2% peptone, and 2% glucose) for at least seven generations until mid-log phase (OD_600_ ~ 1). SILAC yeast cells were grown in SC supplemented with 0.05 mg/ml of L-arginine: HCl (U-13 C6, 97–99%) and L-lysine:2HCl (U-13 C6, 97–99%) (Euriso-top), and 0.2 mg/ml of proline (Sigma). A second culture containing non-labeled amino acids was inoculated in parallel. Cultures were incubated shaking (180 rpm) at 30 °C for at least seven generations until OD_600_ = 1. Light labeled cultures were treated with 0.5 M NaCl for times indicated. For parallel reaction monitoring (PRM) analysis Hog1as cells expressing Kic1-, Orm2-, and Vps53-HB tandem affinity tag fusion proteins were grown to OD_600_ = 1, treated either with DMSO (mock) or 0.25, 0.5, 5 μM as-inhibitor SPP86 (Tocris Bioscience), followed by a 5 min exposure to 0.5 M NaCl.

### HeLa cells growth conditions

HeLa samples [7] were kindly provided by Karl Mechtler. Briefly, cells were harvested, washed with 1 M PBS, suspended in lysis buffer (8 M urea, 50 mM TrisHCl pH 8, 150 mM NaCl, 1 mM PMSF, complete protease inhibitor, benzonase), and subsequently disrupted by sonification. Extracts were cleared by centrifugation (15,000×g, 10 min, 4 °C) and proteins were precipitated by adding 5x excess of 100% ice-cold acetone (Applichem) (overnight, − 20 °C) and pelleted by centrifugation 15,000×g, 30 min, 4 °C). The pellet was re-suspended in 80% ice-cold acetone, centrifuged for 5 min at 15000×g, air-dried for 5 min and subsequently suspended in urea buffer (8 M urea, 0.5 M ammoniumbicarbonate). Soluble proteins were reduced with dithiothreitol (DTT) and alkylated using iodoacetamide (IAA), digested first with Lys-C for 2 h at 30 °C, and then with trypsin overnight at 37 °C. HeLa samples were measured in an HPLC-MS/MS-setup using a Q-Exactive HF-X mass spectrometer (Thermo Fisher Scientific).

### Proteome discoverer original analysis [4]

Data analysis was performed using the SEQUEST algorithm (Proteome Discoverer 1.3 and 1.4) using the Saccharomyces Genome Database (SGD) (version February 2011) along with contaminants derived from common laboratory contaminants database (MQ). Fixed modifications included carbamidomethylation of cysteine, whereas variable modifications encompassed protein N-terminal acetylation, deamidation, oxidation of methionine, phosphorylation of serine, threonine and tyrosine, and heavy labels of arginine and lysine (Arg6, Lys6). Enzyme specificity was set to “Trypsin” and a maximum of 2 missed cleavages per peptide was allowed. For the assignment of phosphorylation sites we integrated the tool phosphoRS into the Proteome Discoverer pipeline, and considered 70% phosphorylation probability as an adequate threshold for phosphorylation site assignment. We performed the SEQUEST analysis against the SGD database, as well as a decoy database (reversed sequences) and calculated an empirical FDR < 1% at the level of peptide spectrum matches (PSMs). Separately, we calculated an FDR at peptide and protein level as well (FDR < 1%). To quantify phosphorylation events accurately, we performed a phosphorylation site group as explained in detail in the section “Phosphorylation site groups”. We considered potential arginine-to-proline conversion by calculating a correction factor based on the SILAC ratio biases observed for peptide groups that are differential in the number of prolines. SILAC Heavy-to-Light ratios were accordingly corrected, log_2_-transformed, and additionally summarized at the level of phosphorylation site groups. More details on the pipeline if required can be extracted from the individual search files deposited at PXD004294 to PXD004300.

### MaxQuant re-analysis

The following MS shotgun datasets published in Romanov et al. [4] were considered for our re-analysis approach: setup SR, setup I + 0′S, setup I + 5′S and setup I + 10′S. MaxQuant (version 1.5.2.8) re-analysis was performed using default parameters, with following features: Saccharomyces Genome Database (SGD) (version February 2011) was used in combination with common laboratory contaminants database (MQ) for peptide spectrum matching. Modifications, such as protein N-terminal acetylation, deamidation of asparagine and glutamine, oxidation of methionine, and phosphorylation of serine, threonine and tyrosine were set as variable, whereas carbamidomethylation of cysteine was set as fixed. A maximum of 5 variable modifications per peptide was allowed. Enzyme specificity was set to “Trypsin/P” and a maximum of 2 missed cleavages per peptide was allowed. Heavy labels (‘Arg6’, ‘Lys6’) were specified, ‘Requantify’ and “Match between runs” was activated. The option to treat leucine and isoleucine as indistinguishable was activated. Computational processing, log_2_-transformation of SILAC ratios and correction for arginine-to-proline conversion was performed as described in [4]. Phosphopeptides were filtered for phosphorylation site assignment probability ≥70% and grouped by phosphorylated residues. The mass spectrometry proteomics data have been deposited to the ProteomeXchange Consortium [8] via the PRIDE partner repository with the dataset identifier PXD011935.

### Phosphorylation site groups

To facilitate interpretation of phosphorylation sites, we grouped peptides together where the same residues are phosphorylated, regardless of potential missed cleavages or additional modifications such as oxidation (corresponding to a so-called “phosphorylation site group”). For each biological replicate, ratios of phosphorylation site groups were calculated as the average of all peptide ratios available in a group. These ratios were then averaged across biological replicates for the final ratio of the phosphorylation site group.

### Mass spectrometry-based screen for probing of phosphorylation kinetics

SILAC-labeled cells were harvested by filtration, immediately deep frozen in liquid N_2_ and suspended in TRIzol reagent (Invitrogen) for protein extraction [4, 5]. Following TRIzol purification [5], proteins were subjected to dithiothreitol (DTT) and iodoacetamide, and tryptic digestion. After desalting on Strata-X 33 μm Polymeric Sorbent (8B-S100-TAK columns, Phenomenex) and drying, peptide carboxyl groups were esterified in methanolic HCl as described in [9]. Esterified peptides were dried, dissolved in 30% ACN / 30% methanol / 40% H_2_O and incubated for 1 h with 40 μl PHOS-Select™ iron affinity resin (Sigma), washed with 0.003% acetic acid, and eluted with 50–125 mM Na_2_HPO_4_ (pH 6.0). Eluates were analyzed on an UltiMate™ 3000 Dual LC nano-HPLC System (Dionex, Thermo Fisher Scientific) coupled to a hybrid linear ion trap/Fourier transform ion cyclotron resonance mass spectrometer (LTQ-FT, Thermo Fisher Scientific), applying settings described previously [4, 5]. The obtained spectra were searched both by SEQUEST in the Proteome Discoverer 1.4 software package (Thermo Fisher Scientific) and MaxQuant 1.5.2.8 against the SGD database (version February 2011) plus contaminants, with similar settings as described above. The data have been deposited to the ProteomeXchange Consortium [8] via the PRIDE partner repository with the dataset identifier PXD011935.

### Poly histidine, biotinylation signal (HB) tandem affinity purifications

HB pull downs were performed as described elsewhere [5]. Cells were harvested by filtration, deep frozen and ground using a SPEX Freezer Mill 6870 (SPEX SamplePrep, Metuchen, NJ, USA) applying standard settings [5]. The cell powder was suspended in buffer 1 (6 M guanidine HCl, 50 mM Tris pH 8.0, 5 mM NaF, 1 mM PMSF, 2 mM sodium orthovanadate 0.1% Tween, protease inhibitor cocktail (Roche, Basel, Switzerland, 11,873,580,001), pH 8) and cleared by centrifugation (13,500×g, 15 min, 4 °C), incubated with Ni2 + −Sepharose beads (GE Healthcare, Buckinghamshire, UK, 17–5318-06) for 4 h at room temperature, washed with urea buffer (8 M urea, 50 mM sodium phosphate buffer pH 8.0 (and pH 6.3), 300 mM NaCl, 0.01% Tween 20). Proteins were eluted in urea buffer pH 4.3 containing 10 mM EDTA, incubated with streptavidin-agarose beads, washed with urea wash buffer containing 1% SDS and without SDS. Beads were re-buffered to 50 mM ammonium bicarbonate (ABC). Samples were reduced using DTT, Cys-residues were alkylated with 20 mM iodoacetamide (IAA), incubated with 300 ng trypsin (Trypsin Gold, Mass Spectrometry Grade, Promega) at 37 °C overnight, quenched with trifluoroacetic acid (0.5% final concentration) and desalted using C18 Stagetips [10].

### PRM analysis

Peptides were separated using a 60 min gradient (HPLC setup as described above). PRM data acquisition was performed using a scheduled method with 6 min windows for each target based on the retention time determined from a prior data-dependent (DDA) LC-MS/MS run (analyzed using Proteome Discoverer as described above) of 5% mock-treated samples. Raw data were obtained on an Orbitrap Q-Exactive HF-X (Thermo Fisher Scientific) mass spectrometer applying the following settings: survey scan with 30 k resolution, AGC 1E6, 30 ms IT, over a range of 380 to 1400 m/z, PRM scan with 30 k resolution, AGC 1E5, 100 ms IT, isolation window of 0.7 m/z with 0.2 m/z offset, and NCE of 27%. Data analysis, manual validation of all transitions (based on retention time, relative ion intensities, and mass accuracy), and relative quantification was performed in Skyline [11]. Up to six characteristic transitions were selected for each peptide and their peak areas were summed up for peptide quantification across charge states (total peak area). Unphosphorylated peptides that were not affected by any phosphorylation event were considered for normalization of the remaining peptides of the same protein. In a second normalization step peptides were normalized to the respective median (normalized) intensity in the mock experiments (in log_10_-space). We applied the standard *t*-test per protein to compare intensities from the mock samples to all inhibitor-treated samples together. PRM data have been deposited to the PanoramaWeb [12] (https://panoramaweb.org/gXvdQ2.url) and to the ProteomeXchange Consortium [8] via the PRIDE partner repository with the dataset identifier PXD013789.

### Gel shift assays

Cells expressing candidate proteins fused to a HKMT-myc tag were grown until mid-log phase, treated with 0.5 M NaCl (final concentration) for 0, 5, 30 and 45 min, respectively, and harvested by centrifugation (2000×g, 1 min). Protein extraction was carried out by glass bead lysis using urea sample buffer (8 M Urea, 300 mM NaCl, 50 mM Tris-HCl pH 8, 50 mM NaPO_4_ pH 8, 0.5% Nonidet P-40). WCLs were mixed with 3x urea loading dye (116 mM Tris-HCl pH 6.8, 4.9% glycerol, 7.99 M Urea, 143 mM 2-mercaptoethanol, 10% SDS, bromophenol blue) and separated on SDS-PAGE gels. Gelshifts were visualized by Western blot using an antibody recognizing myc (4A6, Merck Millipore) or HA (12CA5). Loading was controlled using an antibody recognizing Cdc28 (P7962, Sigma).

### Protein-protein proximity assay (M-track)

M-track assays were performed as described previously [1, 4, 13]. 25 ml cultures of respective M-track yeast strains were grown until mid-log phase (OD_600_ ~ 1), treated with 1 M sorbitol (final concentration) for 40 min, harvested by filtration and immediately deep-frozen in liquid N_2_. Frozen cell pellets were re-suspended in 250 μl ice-cold urea lysis buffer (8 M urea, 0.3 M NaCl, 50 mM Tris/HCl pH 8, 50 mM Na_2_HPO_4_/NaH_2_PO_4_ pH 6.8, 0.5 Nonident P40). Protein extraction was carried out using a FastPrep©-24 homogenizer (mpbio) by 3 cycles of 30 s of bead beating at level 5.5. Whole cell extracts were cleared from insoluble material by two consecutive centrifugation steps (13,500×g, 20 min, 4°). 100 μl of cleared protein extract was mixed with 2x Laemmli buffer (1 M Tris-HCl pH 6.8, 10% SDS, 10% Glycerol, 1 M DTT). Proteins were resolved by SDS-PAGE (8%) and transferred to nitrocellulose in a submerged tank. Blocking was performed overnight using 2% milk in PBS-T. Histone H3 Lysine 9 trimethylation (me3K9H3) of protA-H3-HA tags was visualized using an antibody recognizing me3K9H3 (1:2000 dilution in 1% yeast extract (YE) in PBST, Novus #NBP1–30141). Membranes were incubated with primary antibody for 1 h at 4 °C, followed by 1 h incubation at 4 °C with HRP-conjugated goat anti-mouse (1:5000 dilution in 1% YE in PBS-T, BioRad #170–6516) secondary antibody. No washing steps were performed between primary and secondary antibody incubation. Loading was controlled using an antibody recognizing HA (1:5000 in PBS-T, 12CA5). PicoECL (Thermo Scientific) was used for enhanced chemiluminescent (ECL) detection. Peak areas of me3K9H3 and HA signals were determined by densitometric analysis of scanned Western blot films using ImageJ [14]. Proximity signals were calculated as follows: All signal intensities were log_2_ transformed. Each Western blot experiment contained a three point dilution series of the positive control (Nup2-HKMT). Least squares linear regression was performed independently to the me3K9H3 and HA signals of to the dilution series, which was then used to correct me3K9H3 and HA signals for unequal loading amounts between samples and to normalize between different Western blot experiments. Proximity signals were calculated as the log_2_ ratio of normalized me3K9H3 over HA signal intensity and rescaled by subtracting the mean proximity signal of the negative control (Hog1-protA-H3) and subsequently dividing by the mean proximity signal of the positive control (Nup2-HKMT). We used a one-tailed Welch’s *t*-test to identify the statistically significant candidates. For each candidate, the signal intensities of all replicates were compared against all signal intensities of the negative control (Hog1-protA-H3). *P*-values were corrected for multiple testing by using the Benjamini-Hochberg procedure to generate *q*-values.

### GO enrichment analysis based on yeast GO-slim

Gene ontologies were extracted from the SGD Gene Ontology Slim Mapper (mapping file downloaded in October, 2018), hence broad and high-level GO-terms maintained by the Gene Ontology Consortium (GOC; [15, 16]). MQ-, PD- derived and combined (PD- and MQ-overlap) Hog1-dependent proteins were mapped against the GO-Slim-terms; the background was set as the entire proteome of *Saccharomyces cerevisiae*. The enrichment and respective *p*-value for each GO-term were calculated for all three sets based on the Fisher Exact test implemented in the *scipy* Python package [17]. *P*-values were adjusted using the Benjamini-Hochberg procedure [18]. For further processing and visualization we considered GO-terms in “biological processes” with an adjusted *p*-value ≤0.1 and a hierarchy level ≥ 2.

### Protein network analysis

Protein-protein interaction network for all putative targets of Hog1 was created using STRING database (version 10.5) [19]. All factors listed in Fig. 2d were used as search entries, with first neighbors automatically included in the network by the STRING database. All interaction predictions were based on physical, genetic, and text mining evidence types with the minimum confidence score of > 0.7 (high). Obtained isolated protein networks were then re-analyzed separately and allowed expansion to interactions with a lower minimum confidence score. We then manually curated those additional interactions based on literature updates [20, 21].

### Boxplots

The lower and the upper hinges of the boxes correspond to the 25% and the 75% percentile, and the bar in the box the median. The upper and lower whiskers represent the largest and lowest values, respectively (but at maximum 1.5 times the IQR). Points outside the whiskers are plotted individually. Tests were performed using a nonparametric Mann-Whitney *U*-test.

### Computational methods for comparison of peptide intensities and scores

For all comparisons between the search engines, we generally set all leucines in peptide sequences to isoleucine. We determined whether peptides identified with both search engines, PD and MQ, could be distinguished by their precursor intensity, scores or peptide length from the peptide identifications that were unique to either one of the programs. For each software, we compared the frequency distribution of intensities/scores/peptide lengths from the overlap against the unique set of peptide identifications, respectively. In the case of MQ, the following parameters from the evidence-file were taken into account: (1) *Intensity*, as defined per scan, (2) *Score*, as defined per scan, and (3) *Length*, as defined per peptide. For PD, the following parameters from the PSM-file were taken into account: (1) *Intensity*, as defined per scan, (2) *Xcorr*, as defined per scan, and (3) *peptideLength*, as defined per peptide. To compare the two respective distributions against each other, the Mann-Whitney *U*-test was used.

## Introduction

Living cells integrate various physico-chemical stimuli via complex intracellular signaling systems, involving highly intertwined kinase- and phosphatase networks. Numerous studies aimed to unravel such signaling networks using quantitative mass spectrometry (MS)-based proteomics, often with the focus to comprehensively identify substrates of a given kinase or phosphatase at a specific condition [4, 5, 22–27]. To achieve this goal, dynamic changes in the phosphorylation status of thousands of sites are monitored across different experimental conditions. The analysis of quantitative MS data requires sophisticated bioinformatic processing of the raw data to ensure valid and comprehensive interpretation. Several quantitative proteomics software tools have been developed [28–33] applying different algorithmic pipelines, which may or may not lead to different outcomes on peptide identification and quantification. Two commonly used software packages for the quantitative analysis of SILAC (stable isotope labeling with amino acids in cell culture) MS data are Proteome Discoverer™ (PD) [Thermo Fisher Scientific] and MaxQuant (MQ) [29]. A major complication for integrative analysis of multiple datasets is the low overlap of quantified phosphopeptides. This limited overlap poses a major problem in the field, which caused researchers to improve software for data analysis and develop novel technological strategies, like e.g. BoxCar [34] or data independent acquisition methods such as SWATH MS [35]. While the choice of software for analyzing a specific dataset is mainly subject to the technical and methodical constraints of the experiment as well as research group conventions, it stands to reason that the use of a broader range of tools might increase proteome coverage and consequently lead to new biological insights [36–38].

Here we re-analyzed an extensive, quantitative MS-based phosphoproteomics dataset [4], previously published by our group, with an alternative software package [29, 33]. This dataset, designed to identify substrates of the mitogen-activated protein kinase Hog1, a p38 homolog and key regulator of the high osmolarity glycerol pathway in *Saccharomyces cerevisiae* [4], comprises 204 individual LC-MS runs, integrates four different experimental conditions and led to the identification of more than 30 substrate proteins of the MAPK [4]. In comparison to the original analysis with PD (versions 1.3 and 1.4) using the database search engine SEQUEST, our reanalysis with MQ (version 1.5.2.8) resulted in only minor differences in quantification of SILAC ratios. However, the number of identified phosphorylation sites when integrating the results of both programs was much larger. In total, 15 previously unidentified putative substrates and numerous indirect targets of Hog1 were revealed. Identified target proteins were further validated by their ability to directly interact with Hog1 in vivo [1, 4, 13]. Ultimately, our comparative analysis increased the number of identified Hog1-substrates by roughly 30%.

## Results

### Combined use of two proteomic quantitative software packages increases coverage

**Fig. 1:**
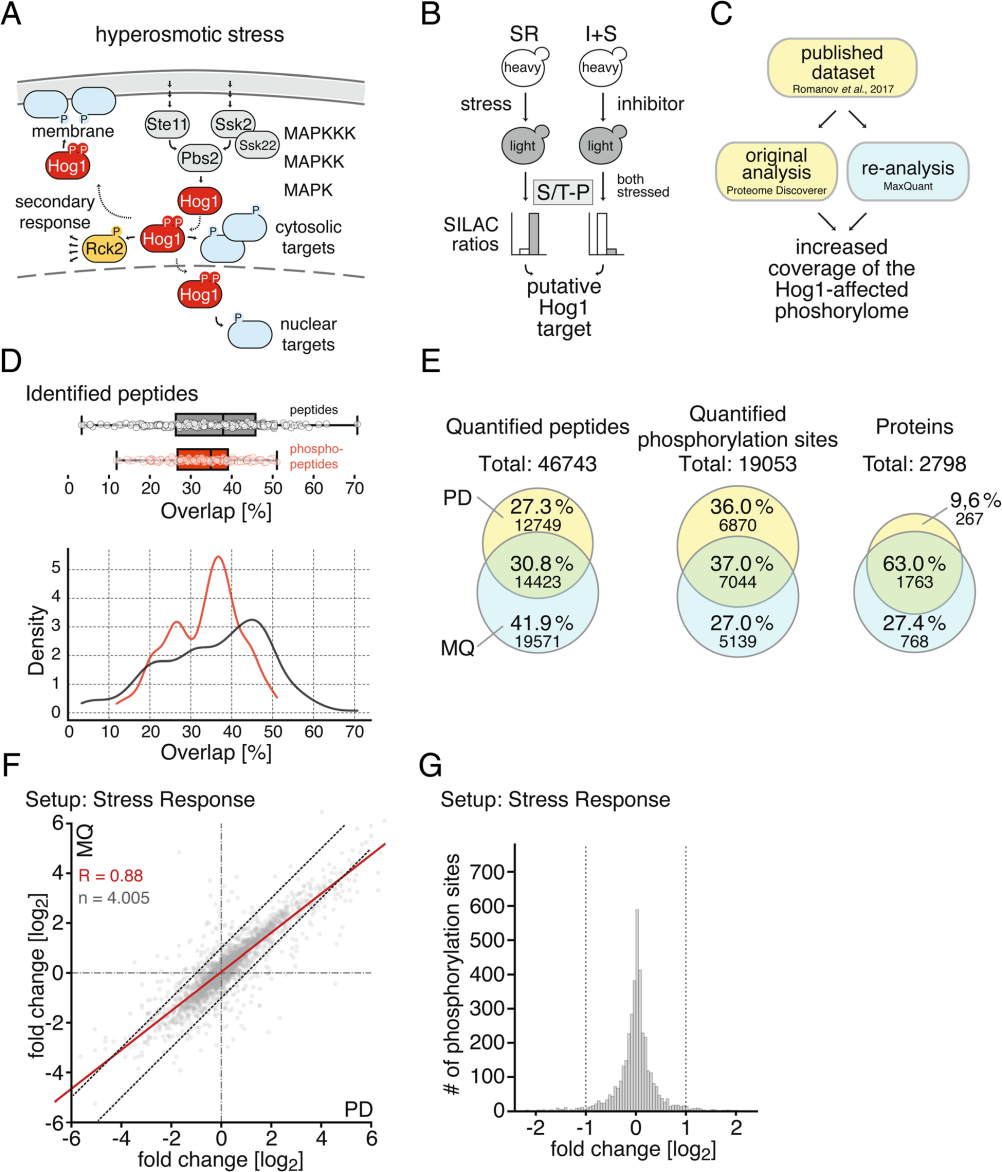
**a** Cartoon illustrating the HOG pathway. Its central module consists of the MAPK Hog1, the MAPK kinase (MAPKK) Pbs2, and the three MAPKK kinases (MAPKKK) Ste11, Ssk2, and Ssk22. Upon activation by extracellular hyperosmolarity, Hog1 coordinates the osmostress response by phosphorylating its target proteins. Ultimately, the cascade leads to the activation of downstream kinases, such as Rck2. **b** Illustration of experimental conditions from Romanov et al., 2017 [4]. **c** Illustration of study concept. **d** Box- and density plot showing the degree of overlap in % of identified peptides (grey) and phosphorylated peptides (red) between PD and MQ outputs for each raw file (dot). Black line in box plot indicates median overlap (**e**) Venn diagrams showing percentage and total number of quantified peptides, quantified phosphorylation sites and proteins identified by MQ (light blue), PD (yellow) or both (green). **f** Correlation of SILAC log_2_-ratios of mutually quantified phosphorylation sites of setup SR. Lines indicate limits of +/− 1 quantification difference (**g**) Histogram displaying quantification difference calculated as MQ/PD SILAC-ratio [log_2_] of quantified phosphorylation sites of setup SR. Lines indicate cut-off (+/− 1 quantification difference)

System-wide characterization of signaling networks is commonly addressed via MS-based proteomics approaches. The high osmolarity glycerol (HOG) response in *Saccharomyces cerevisiae* serves as a paradigm of complex signaling networks. Elevated extracellular osmolarity results in activation of the mitogen-activated kinase (MAPK) Hog1 which in turn propagates the stress signal by phosphorylating target proteins at specific motifs, characterized by a serine or threonine followed by a proline (S/T-P) (Fig. 1a).

We have recently conducted an extensive proteomics study with the aim to comprehensively identify direct substrates of Hog1 [4]. Global changes in the yeast phosphorylome were quantified in response to hyperosmotic stress (setup SR). Additionally, we analyzed the effect of Hog1-inhibition (in comparison to mock treatment) in hyperosmotically challenged cells to determine the impact of the active MAPK (setups I + 0′S, I + 5′S and I + 10′S, see [4]) (Fig. 1b). SILAC labeling was used in combination with TiO_2_-based phosphopeptide enrichment and strong cation exchange (SCX) fractionation to enable in depth analysis of the phosphorylome (Additional file 1: Figure S1A). The dataset comprises 204 individual LC-MS runs, which were acquired on Thermo Velos Orbitrap and Thermo Q-Exactive instruments in a data-dependent acquisition (DDA) mode. Raw data were analyzed with PD (versions 1.3 and 1.4) using SEQUEST for identification of peptides from MS2 spectra. A standard target-decoy approach was employed to determine the false discovery rate on spectrum and protein level (FDR cut-off < 1%).

Phosphorylated S/T-P motifs that increased in abundance in response to stress and showed sensitivity to inhibitor treatment were considered putative Hog1-substrates (Fig. 1b). It is important to clarify here that most stress-induced S/T-P sites are not impacted by the kinase at all. Out of ~ 200 proteins that were affected by hyperosmotic stress, only around 30 proteins were found to harbor proline directed kinase motifs (or the alternative S/T-S/T-P motif) that displayed Hog1-dependent phosphorylation behavior [4], including eight previously described Hog1-substrates. However, several hallmark phosphorylation sites of Hog1 were not covered, which might be due to the inherent incompleteness of MS shotgun approaches. We speculated that not all spectra recorded in this dataset resulted in successful peptide identification or quantification by the applied data analysis workflow and that re-analysis with alternative software might recover complementary information and thereby potentially increase the coverage of the Hog1-affected phosphoproteome.

We therefore re-analyzed the MS dataset (setups SR, I + 0′S, I + 5′S and I + 10′S) by Romanov et al. using MQ (see Methods; Fig. 1c) and integrated the results with the PD-derived results described in [4]. Specifically, we determined the degree of overlap of identified peptides between PD and MQ outputs for each raw file (Fig. 1d) and observed overlaps ranging from ~ 5 to 70% with a median of 37.9%. We examined whether different parameters such as MS signal intensity, peptide-spectrum-match (PSM)-score, or peptide length might contribute to the different coverage obtained with PD and MQ. In general, both packages show a similar performance with the exception that low scoring PSMs are better covered by MQ, probably stemming from differences in target-decoy approach-based FDR-estimation between the software packages (Additional file 1: Figure S1B-C). The level of overlap is even lower (median 34.9%) when solely considering identified phosphopeptides. Ultimately, we obtained a total overlap of 30.8% for all quantified peptides (Fig. 1e).

To facilitate phosphorylation site analysis, we integrated peptides where the same residues are phosphorylated (regardless of potential missed cleavages or additional modifications) to a so-called “phosphorylation site group” (PSG), similarly to procedures described in Romanov et al. [4]. The eventual ratio for the phosphorylation site group is an average over all peptide ratios available in the group across all biological replicates (see Methods), hereafter referred to as “phosphorylation site”. A total of 19.053 phosphorylation sites could be quantified when considering all MS data files and both software packages (Fig. 1e). Both tools commonly identified only 37.0% (7044) of all quantified phosphorylation sites (Fig. 1e). Each data analysis platform added a roughly equal share of unique quantifications, namely 36.0% (6870 quantified phosphorylation sites) from PD and 27.0% (5139) from MQ. Notably, the overlap between the two search engines rises to 63.0% when comparing protein instead of phosphorylation site coverage (Fig. 1e). This difference in overlap is presumably due to proteins harboring more than one affected phosphorylation site, which could be quantified individually by either software. Taken together, re-analyzing the raw data extended the number of quantified phosphorylation sites by one third.

To determine reproducibility of quantification between PD and MQ, SILAC ratios of phosphorylation sites of setup SR covered by both programs were compared (Fig. 1f). We observed a high level of correlation (*R* = 0.88), suggesting that both software tools almost equally quantify phosphorylation sites. When considering the MQ/PD-ratio for commonly quantified phosphorylation sites 93.43% were within a limit of +/− 1 (log_2_) quantification difference (Fig. 1g). Similar observations were made with the other setups (Additional file 1: Figure S1E-J). We therefore conclude that the degree of divergence in quantification of the two softwares is negligible and phosphorylation site-quantifications covered in only one of the two datasets can be considered valid.

### Identification of novel direct Hog1-substrates

**Fig. 2:**
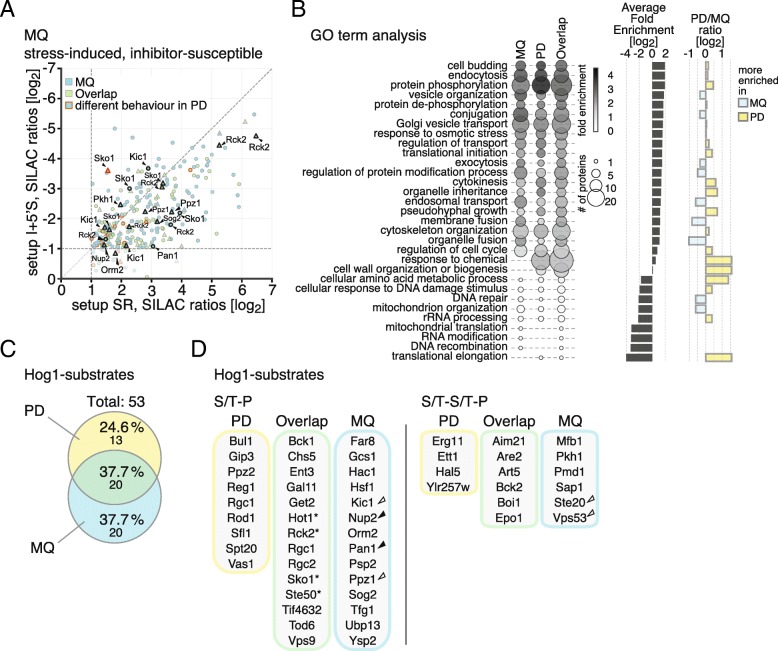
**a** Scatter plot displaying SILAC ratios of setups SR (*x*-axis) and I + 5′S (*y*-axis). S/T-P motifs: triangles. Other motifs: circles. Ratios are log_2_-transformed. Similar analyses were made with setups I + 0′S and I + 10′S, respectively (not shown). **b** Results from gene ontology (GO) enrichment for three sets of Hog1-dependent phosphorylation sites derived from MQ, PD, and both search engines. GO-terms were filtered to have at least one *q*-value ≤0.1 in either set, allowing hierarchical levels ≥2 and solely “biological processes” as a GO category. The bubble size corresponds to the number of proteins associated with a given term; the color corresponds to the fold enrichment. The GO-terms were sorted according to the average fold-enrichment (side bar plot). On the right-hand side the PD/MQ-ratio between the respective enrichments are shown as a bar plot. In case the ratio is ≥1 (signified by red dashed line), the enrichment of the corresponding term is higher in PD vs. MQ (yellow coloring), and vice versa (blue coloring). **c** Venn diagram showing percentage and total number of stress- and Hog1-induced S/T-P motifs. Light blue: MQ, yellow: PD, green: overlap. **d** Putative Hog1-target proteins identified via genuine S/T-P (left) or S/T-S/T-P (right) motifs. Color-coding similar to (**c**). Filled arrowheads: known Hog1-target proteins in MQ-derived dataset. Open arrowheads: candidates that did not qualify as Hog1-substrates in [4] due to lack of overlap between experimental setups. *: alternative phosphorylation sites found with PD or MQ

To identify Hog1-substrates, we focused on phosphopeptides that increase in abundance in response to high osmolarity but remain static if the MAPK is inhibited (Fig. 2a, Additional file 6 Table S1). The phosphorylation status of these sites is directly or indirectly dependent on Hog1-activity. Whereas 204 phosphorylation sites with such behavior were identified using PD [4], re-analysis with MQ resulted in 273 Hog1-dependent phosphorylation sites. Of those, 101 phosphorylation sites were covered by both tools. These numbers add up to a total of 172 stress- and Hog1-dependent sites uniquely identified by MQ and 103 by PD, enhancing the size of the Hog1-dependent phosphorylome [4] by 36.0%.

To capture cellular processes affected by Hog1-activity, we performed a gene ontology (GO)-term analysis using the Hog1-dependent phosphorylation sites derived from the MQ- and the PD-based analysis, and a combination of the results (Fig. 2b and Additional file 7 Table S2). GO-terms derived from both search results were highly similar (*R* = 0.83 for fold enrichments), with these phosphorylation sites found to be associated with signal transduction (such as protein phosphorylation/dephosphorylation, response to osmotic stress etc.), cell cycle regulation, endocytosis, transport- and cytoskeleton-related processes, which is in line with the general understanding of the HOG response [4, 39, 40]. Besides some other connections to translational initiation and cell budding, membrane-associated processes (adjusted *p*-value = 2.25 × 10^− 3^) were overrepresented in the MQ-derived dataset, such as conjugation, membrane and organelle fusion, as well as organelle inheritance and exocytosis, ultimately giving a potentially novel context to Hog1 signaling.

We next tested whether the list of putative direct Hog1-substrates was extended by the integration of results from both programs. For this purpose we selected sites phosphorylated at S/T-P motifs and, in addition, sites phosphorylated at S/T-S/T-P motifs, in order to prevent omission of targets due to incorrectly localized phosphorylation sites. 49 S/TP (or S/T-S/T-P) motifs (40 proteins) were found within the MQ-derived set of stress- and Hog1-dependent phosphorylation sites (Fig. 2c). 28 of these phosphorylation sites, corresponding to 20 proteins, have not been covered in the PD-based analysis [4] (Fig. 2d). Seven of the 40 proteins are known substrates of the MAPK, namely the transcription factors Hot1 and Sko1 [41, 42], the nucleoporin Nup2 [43], the endocytotic factor Pan1 [5], the serine/threonine protein phosphatase Ppz1 [4], the MAPKAP kinase Rck2 [44], and the protein kinase regulator Ste50 [27]. Furthermore, Hot1, Rck2, Sko1 and Ste50 were also covered in the PD-derived dataset [4], however, with alternative Hog1-dependent phosphorylation sites (Additional file 6: Table S1).

Among the MQ-derived, newly identified Hog1-affected proteins, we found interesting factors such as the p21-activated kinase Kic1 [45, 46] and Orm2, a protein linked to TORC1/2- and Ypk1-mediated sphingolipid homeostasis [47–49]. Although two phosphorylation sites of Kic1 showed stress-responsiveness (Thr^625^) or susceptibility to Hog1-inhibition (Ser^723^) [4] Kic1 did not qualify as a target due to the lack of overlap between setups in the original PD-analysis. Re-analyzing the raw data with MQ confirmed the stress- and Hog1-dependency of Kic1 (Thr^625^ and Thr^1073^). A similar scenario occurred with Orm2 and Ppz1 both of which did not have sufficient coverage in the PD-analysis; the re-analysis, however, suggests a Hog1-mediated phosphorylation of Thr^18^ of Orm2 and Ser^265^ of Ppz1 (Additional file 6: Table S1). The latter has been previously identified as a Hog1-substrate, based on Hog1-dependent phosphorylations of its paralogue Ppz2 and its ability to interact with the kinase [4].

In total, our combined analysis revealed 15 novel putative Hog1-target proteins (10 via genuine S/T-P motifs), namely the kinases Kic1, Pkh1 and Ste20, the transcription factors Hac1, Hsf1 and Tgf1, the retrograde transport-associated proteins Gcs1, Vps53 and Ysp2, the mitochondria-associated proteins Mfb1 and Psp2, and the ubiquitin-specific protease Ubp13. Further putative targets include Far8 - a protein involved in the recovery from cell cycle arrest, Orm2 - a protein involved in sphingolipid homeostasis, Sog1 - a key-component of the RAM signaling network (and binding partner of kinase Kic1), and finally, Pmd1 and Sap1, two proteins of unknown function (Fig. 2d, Additional file 6: Table S1**)**.

### Phosphorylation kinetics as a proxy for Hog1-dependency

Before proceeding with further functional conclusions, it is arguably important to examine Hog1-dependency of phosphorylation events using orthogonal means. We assumed that measuring a respective phosphorylation kinetic read-out might provide such evidence to validate Hog1-dependency. This is largely based on our recent report on different stress-induced phosphorylation kinetics at two S/T-P motifs of the early endocytosis factor Pan1, namely Ser^1003^ and Thr^1225^ [5]. Specifically, Pan1 Thr^1225^ becomes directly phosphorylated by Hog1 in response to elevated extracellular osmolarity and shows transient phosphorylation kinetics. Phosphorylation at the Hog1-independent S/T-P motif Ser^1003^, on the other hand, continuously increases until a maximum is reached 30 min after stress induction.

**Fig. 3:**
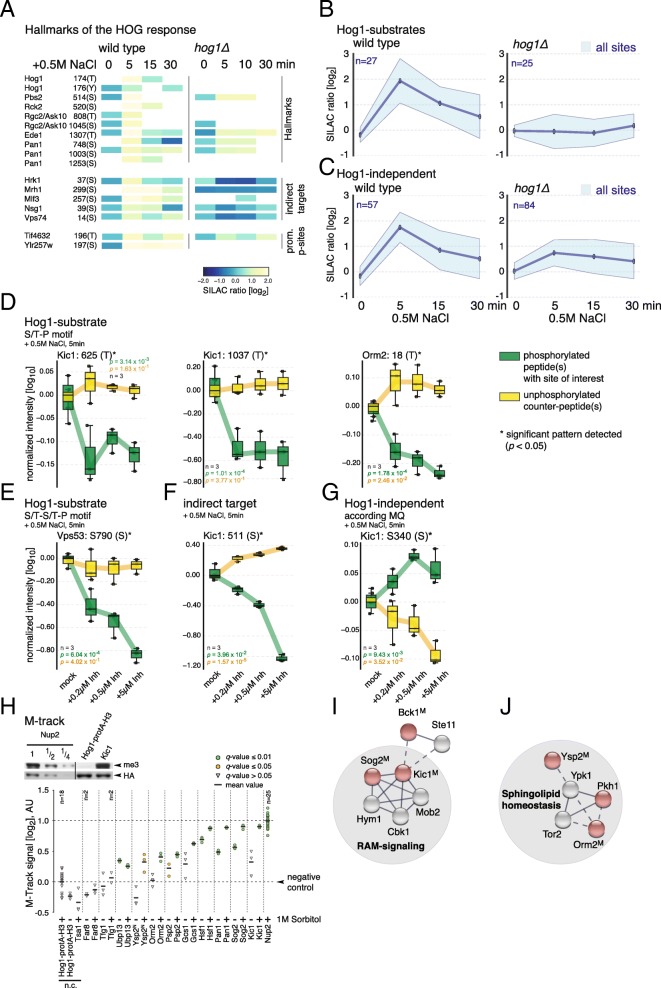
**a** Heatmap showing SILAC ratios of selected phosphopeptides at 0, 5, 15 and 30 min after treatment with 0.5 M NaCl. Hallmarks: well-known phosphorylation events of osmostress signaling. Indirect targets: stress-inducible and inhibitor-susceptible phosphopeptides phosphorylated at non–S/T-P motif sequences [4]. Promiscuous p-sites: phosphorylation sites targeted by multiple kinases. **b** and **c** Average stress-induced phosphorylation kinetics of Hog1-dependent (above) and Hog1-independent (below) phosphorylation sites in a wild type and *hog1*Δ strain. **d-g** Illustration of PRM-measured phosphorylation patterns for Hog1-dependent and -independent sites upon hyperosmotic stress (+ 0.5 M NaCl) and inhibitor treatment (SPP86). N (biological replicates) = 3. For a given phosphorylation site, the green box plots represent the (mean) normalized intensities for the respective phosphopeptide(s). The yellow box plots illustrate the normalized intensities for unphosphorylated counter-peptides. Significance was assessed by comparing intensities derived from all pooled inhibitor-treated samples with those from the mock sample (*t*-test, *p* < 0.05). **h** Above: Representative Western blot showing M-track protein protein proximity signals obtained for Kic1. Hog1-protA-H3: background control, Nup2: positive control. Below: Proximity signals. *n* = 3 replicates per sample except when indicated differently. Ratios are log_2_-transformed. Black lines indicate average proximity signal. Proximity signals that differ significantly from background are marked in green (*q* ≤ 0.01) and orange (*q* ≤ 0.05 and > 0.01) filled circles. Grey filled triangles: *q* > 0.05. ^N^: N-terminal HKMTmyc fusion. **i** and **j** Newly identified Hog1 network hubs based on STRING. Red filled circles: putative target proteins identified in this study. ^M^: positive M-track signal. Gray circles: first neighbor according to STRING. Shaded circles enclosing groups of proteins highlight functional groups. Filled lines indicate high, dashed lines confidence score ≤ 0.4 according to STRING

We therefore implemented seven additional experimental MS setups with 2–3 replicates for a reasonably fast probing of the kinetics of a large number of phosphorylation sites. In a quantitative SILAC-MS setup without prior SCX-fractionation (see Methods), global changes in the phosphorylation pattern of wild type cells at 0, 5, 15 and 30 min after exposure to increased salt concentrations were measured. This setup adequately reflected the response of cells challenged with hyperosmolarity as demonstrated by the phosphorylation kinetics of the key residues of Hog1 (Thr^174^ and Tyr^176^), Pbs2 (Ser^514^), and Rck2 (Ser^520^) [44] (Fig. 3a, Additional file 8: Table S3). Other well known phosphorylation events in osmostress signaling, such as at Thr^808^ of Rgc2 [24], Ser^748^, Ser^1003^ and Ser^1253^ of Pan1 [5], and Ser^1307^ of Ede1 [5], also show consistent phosphorylation patterns [4] (Fig. 3a, Additional file 3: Figure S3A and B). We generally observed the kinetics of the quantified phosphorylation sites to be in good compliance with the extensive phosphoproteomics dataset used for the re-analysis approach (Additional file 8: Table S3).

We next grouped stress-induced phosphorylation sites in Hog1-dependent and Hog1-independent sets according to our PD-and MQ-derived quantifications. The average phosphorylation kinetics profiles were similar between these sets, with Hog1-dependent sites showing a slightly higher maximum at 5 min and lower basal levels at 30 min after stress induction (Fig. 3b). These transient kinetics were apparent for sites, such as Ser^520^ of Rck2 - a main hub regulating the secondary response of Hog1 [4] - as well as for indirect substrates of Hog1 (Fig. 3a, Additional file 3: Figure S3A). However, we also observed sites in this set that became persistently phosphorylated in response to stress, such as the putative direct Hog1-substrate sites Thr^196^ of Tif4632 or Ser^197^ of Ylr257w. Interestingly, these phosphorylation sites have also been assigned to kinases other than Hog1 [50], which might affect the phosphorylation kinetics in response to stress. To corroborate these results we also determined stress-induced phosphorylation kinetics in a *hog1*∆ strain. As expected, the stress-induced phosphorylation of Hog1-dependent sites was diminished, whereas almost all Hog1-independent sites remained phosphorylated in this strain background, albeit with altered kinetic profiles due to missing feedback regulation (Fig. 3a, b, Additional file 2: Figure S2A and B).

The difference between the stress-induced phosphorylation kinetics of Hog1-dependent and -independent sites also became apparent in our additional analysis of published data. Recently, Kanshin et al. examined global properties of the immediate (≤ 1 min) HOG signaling response and defined distinct clusters according to phosphorylation kinetic profiles [22]. Applying these clustering categories on the MS dataset by Romanov et al. we found that ~ 20% of the Hog1-dependent phosphorylation sites (covered in both studies, Romanov et al. and Kanshin et al.) reach a maximum within 1 min (cluster 6), ~ 40% show a delayed sigmoid response curve (cluster 4), while ~ 40% are static, suggesting that these sites become phosphorylated after 1 min of stress induction. Hog1-independent phosphorylation sites, however, show a different distribution with a substantially higher proportion of static sites (84.5%) according to Kanshin et al. (Additional file 2: Figure S2C and Additional file 6: Table S1).

To test whether the newly MQ-identified putative Hog1-targets indeed follow transient phosphorylation kinetics we first performed gel mobility shift assays. However, of all tested candidates (12) only Kic1 and Vps53 showed a subtle decrease in gel mobility at later time points of the osmostress response (Additional file 3: Figure S3A). Orm2, on the other hand, showed a Hog1-independent, transient increase in gel mobility at 5 min after stress induction, indicating a decreased net phosphorylation (Additional file 3: Figure S3A, B and C). Incidentally, Ypk1, the upstream kinase of Orm2, becomes de-phosphorylated at its key regulatory site Thr^662^ [48, 51] upon elevated extracellular salt stress, probably affecting Hog1-independent phosphorylation events on Orm2 as well.

Our computational and experimental analysis thus solidifies the argument that phosphorylation kinetics could indeed be leveraged to define Hog1-dependencies, despite its occasional ambiguity due to promiscuous sites following different response patterns. To gain a more clear-cut and interpretable kinetic signal, however, both the experimental conditions and methodology should be adjusted. Following the kinetic patterns upon inactivation of the MAPK, for example, would arguably provide a more specific indication for local Hog1-dependencies. The methodological context, on the other hand, would require an approach where (sub-stoichiometric) kinetic patterns are captured independently of net phosphorylation effects. Given these criteria, a targeted MS approach was deemed appropriate to provide an accurate fingerprint of phosphorylation kinetics as a function of hyperosmotic stress and Hog1as-inhibitor susceptibility.

Specifically, we compared inhibitor susceptibility by measuring dose-response curves of four different representative types of phosphorylation sites. We focused on i) putative direct Hog1-target sites (Thr^625^ and Thr^1073^ of Kic1 and Thr18 of Orm2), ii) S/T-S/T-P motifs (Ser^790^ of Vps53), iii) putative indirect target sites (Ser^511^ of Kic1), and finally iv) on phosphorylation sites that were differentially quantified by PD and MQ (Ser^340^ of Kic1). Hog1as cells expressing Kic1, Orm2, and Vps53 fused to a HB tandem affinity tag were treated with DMSO (mock) or 0.25, 0.5, 5 μM as-inhibitor (SPP86), respectively, followed by a 5 min exposure to increased extracellular salt concentrations (similar to the experimental setup I + 5′S [4]). The resulting phosphorylation patterns were analyzed using parallel reaction monitoring (PRM). We detected strong inhibitor susceptibility for Thr^1073^ of Kic1 (~ 3-fold), Ser^790^ of Vps53 (~ 6-fold, Additional file 3: Figure S3D), and Thr^18^ of Orm2 (~ 1.7-fold, Fig. 3d and Additional file 6: Table S1) at low inhibitor-concentrations, validating these proteins as putative direct substrates of the MAPK (Fig. 3d and e, Additional file 9: Table S4). The PRM-technology allowed to distinguish the behavior of five different phosphorylation sites on one phosphorylated peptide of Orm2 and to narrow down the target site to Thr^18^ (Additional file 3: Figure S3E). Ser^511^ of Kic1 - a putative indirect target site - showed strong sensitivity to higher inhibitor-concentrations (Fig. 3f), whereas phosphorylation at two adjacent sites, Ser^509^ and Ser^512^, showed different inhibitor dose-response behavior (Additional file 3: Figure S3F). Ser^512^ remained unaffected by inhibitor treatment, confirming that this site responds to stress independent of Hog1. Ser^509^, which was found stress-responsive (~ 8-fold) in our MQ-derived re-analysis dataset, was weakly affected (~ 1.6-fold) by higher inhibitor-concentrations, confirming previous observations (Additional file 6: Table S1 and [4]). Ser^340^ of Kic1 was not (~ 1.2-fold) affected by inhibitor treatment clearly rendering it Hog-independent (Fig. 3g). PRM analysis of Thr^625^ of Kic1 has been compromised by various co-eluting phosphorylated peptide isoforms (a problem that was partly also true for Thr^18^ of Orm2, Additional file 4: Figure S4A), resulting in no conclusive quantification (~ 1.4 fold compared to 2.1 down-regulation in MQ re-analysis, Fig. 3d, Additional file 4: Figure S4B and C and Additional file 6: Table S1). In summary, results from our targeted MS-approach strongly corroborated hypotheses on Hog1-dependencies derived from the MQ-analyzed shotgun results (see Additional file 6: Table S1). Putative direct S/T-P (and probably also S/T-S/T-P) motifs generally show strong susceptibility to inhibitor treatment, whereas indirect sites responded to higher inhibitor-concentrations.

### Validation of Hog1-substrate interactions

To confirm whether the candidate proteins harboring stress- and Hog1-dependent S/T-P motifs directly interact with Hog1, we performed M-track protein-protein proximity assays [1, 4, 13]. Briefly, this assay is based on enzymatic tagging of a histone H3-moiety (designated protA-H3) fused to Hog1. Putative target proteins, where the phosphorylation site could be clearly assigned to a genuine S/T-P motif, were fused to the enzymatic domain of the histone lysine methyltransferase SUV39 (HKMTmyc) and served as bait. We created functional HKMTmyc tag fusions for 12 of the candidates described above, namely: Far8, Gcs1, Hsf1, Kic1, Nup2, Orm2, Pan1, Psp2, Sog2, Tgf1, Upb13 and Ysp2 (the latter as C- as well as N-terminal fusion tags). Upon close proximity to the kinase, the HKMT domain of bait proteins catalyzes tri-methylation of the H3-moiety. Our analysis further included an HKMTmyc-fusion of the cytosolic thioredoxin peroxidase Tsa1 as a negative control. Background signal intensity was defined using a yeast strain expressing only Hog1-protA-H3. Proximity signals were detected by Western blotting using an antibody directed against triple-methylated lysine 9 of histone H3 (me3K9H3). Except Far8, Tgf1 and the background control Tsa1, all tested candidates showed proximity signals significantly above background upon stress treatment. Moreover, 8 of the 12 tested kinase-substrate interactions showed induction of the proximity signal after stress treatment (Fig. 3h and Additional file 5: Figure S5).

In conclusion, we were able to confirm that the majority of the putative Hog1-substrates, identified by our combined analysis using two alternative MS-analysis tools, directly interact with the MAPK. We therefore conclude that our approach indeed could improve the results by increasing the depth of the quantitative MS-data analysis, thus demonstrating the great potential that lies in revisiting published large-scale MS datasets.

## Discussion

In this report we present a combined analysis of an extensive quantitative phosphoproteomic MS-shotgun dataset using two widely used MS software tools, in order to comprehensively capture substrate proteins of the MAPK Hog1. Besides the Hog1-targets derived from the original analysis with PD, a re-analysis with MQ resulted in 15 additional putative substrates of Hog1, that have not been previously associated with HOG signaling. Given that the combination of results from multiple programs could potentially increase the number of false positives, we validated potential targets using a protein-protein proximity assay. The newly identified proteins are therefore most likely genuine substrates of Hog1.

### What potential lies in re-analyzing MS-data?

The idea of re-analyzing published datasets is not new and widely used in the genomics field [52–54]. For proteomics data, on the other hand, major initiatives such as the ProteomeXchange Consortium [55, 56] pave the way for streamlined submissions and dissemination pipelines of proteomics data. Extensive MS-shotgun datasets are constantly published and the corresponding raw data are made available through data repositories, such as the PRIDE or the MassIVE repository [8, 57]. Such datasets are a valuable, yet under-used, resource. The PRIDE repository allows for detailed inspection of post translational modifications of single proteins [58, 59], whereas the MassIVE repository [57] makes it possible to re-evaluate extensive datasets using MS-GF+. These features, however, are usually only available for complete submissions, which constitute only a fraction of all submitted datasets. Furthermore, automated re-analysis is restricted to spectrum identification because quantitative analyses are more complex and require detailed knowledge about the experimental conditions and design.

We picked up this concept and adapted it to our scientific question regarding the quantitative investigation of the Hog1-dependent phosphoproteome. Somewhat surprisingly we observed only a limited overlap between MQ- and PD-derived results, and a roughly equal share of uniquely quantified phosphorylation sites added by each software. We speculate that the difference might be due to the different scoring algorithms (Andromeda vs. SEQUEST) but also to differences during pre-processing steps, including MS1 peak picking, MS1 m/z re-calibration and MS2 precursor re-evaluation. To exclude dataset-specific effects, we analyzed an MS test run of an HeLa cell extract with MQ (version 1.5.2.8) and PD with SEQUEST as search engine (PD version 1.4), and obtained a similarly low level of overlap (Additional file 1: Figure S1K and Additional file 10: Table S5). We therefore conclude that the differences in the output of MQ and PD are indeed caused by differences in the processing steps inherent to each software package and search algorithm. However, our study was not aimed at providing a detailed comparison of the packages, but rather to leverage potential differences to confirm potential targets that would have remained poorly quantified otherwise.

### Are the newly identified candidate proteins genuine targets of Hog1?

Our MQ-based re-analysis of the dataset revealed several Hog1-signaling hallmark phosphorylation sites that have been missed in the original search based on PD, such as Ser^360^ of Hot1 [41], Thr^361^ of Nup2 [43], Thr^1225^ of Pan1 [5], Ser^108^ and Thr^113^ of Sko1 [42] and Thr^341^ of Ste50 [60, 61], confirming the validity of our approach. We additionally recovered phosphorylation sites that did not qualify as a target of Hog1 due to the lacking overlap between setups in the original PD-analysis, such as Thr^1073^ of kinase Kic1, Thr^18^ of Orm2 and also Ser^265^ of Ppz1. In summary, the set of direct Hog1-target proteins could be extended to 53, with 15 novel putative substrates of the MAPK presented here for the first time.

Interestingly, we also found several sites previously connected to Cdc28-mediated signaling [50, 62], to be phosphorylated in a stress- and Hog1-dependent manner, indicating that these sites might constitute an integrative hub for different signaling pathways. This set includes Ser^161^ of Gcs1, Ser^265^ of Ppz1, Ser^304^ of Psp2, Ser^94^ of Sko1, and Ser^546^ of Ste20 (Additional file 11: Table S6). In general, we observed promiscuous phosphorylation of many Hog1-dependent phosphorylation sites when comparing datasets from different studies [4, 50, 62] or from the PhosphoGRID database [63], indicating that some regulatory functions of Cdc28, which are also required for hyperosmo-adaptation, might be compensated by the MAPK, indicating that the MAPK might compensate for some regulatory functions of Cdc28 which are also required for hyperosmo-adaptation [64, 65] (Additional file 11: Table S6).

The fact that almost all candidates selected for an interaction study provided positive signals with Hog1 strongly supports our notion that they constitute genuine targets of the kinase. A comprehensive list of Hog1-substrate sites based on this and previous studies [4, 41, 43, 50, 60–62, 65–76] is provided in Additional file 11: Table S6.

### Novel insights into the Hog1-mediated osmotic stress response of yeast

Our findings regarding Kic1 and Sog2 highlight a previously unrecognized interconnection between the HOG and RAM (regulation of Ace2 activity and cellular morphogenesis) signaling network, which coordinates cell separation in *Saccharomyces cerevisiae* [77–82] (Fig. 3i). Both factors constitute generally conserved [83], regulatory components of the pathway [84]. The impact of Hog1 might therefore extend via RAM to cytoskeletal and actin cortical patch organization, and cell morphogenesis in general, as our GO-analysis suggests. Our combined analysis also allowed the identification of Orm2 as a direct target of Hog1. This evolutionary conserved protein [85] is crucial for coordinating lipid homeostasis [86, 87] and is responsive to ER- and heat-stress in yeast. Upstream of Orm2, kinases Pkh1 and Ypk1 provide a sensor- and feedback loop, which ultimately leads to phosphorylation of Orm2 (at Ser^46^, Ser^47^ and Ser^48^) and release of Orm2-mediated inhibition of sphingolipid biosynthesis in response to heat stress [21]. Here we describe Hog1-dependent phosphorylation of Thr^18^ of Orm2, a site located on the N-terminus adjacent to the three nested consensus Ypk1 phosphorylation sites, and, interestingly, also of Ser^513^ of kinase Pkh1. Generally, disturbance of sphingolipid homeostasis has been shown to directly affect the generation of diverse pathological phenotypes in both, yeast and mammalians [85]. For example, Orm-like protein (ORMDL)-regulated cellular levels of sphingolipids have been associated with several diseases related to chronic inflammation [88] such as rheumatoid arthritis [89], diabetes type 1 [90, 91], and human childhood asthma [92–96].

In addition, we identify a second substrate of the TORC2-activated kinase Ypk1 as putative Hog1 substrate, namely the StARkin domain-containing protein Ysp2, which mediates sterol distribution between plasma membrane and endoplasmic reticulum [20]. In our previous publication we already reported that two additional enzymes involved in ergosterol metabolism are directly targeted by Hog1, the acyl-CoA:sterol acyltransferase Are2 and lanosterol 14-a-demethylase Erg11 [4]. Taken together, these findings strongly point towards several connections of HOG-signaling and the regulation of the membrane fluidity and permeability (Fig. 3j) and could therefore provide interesting insights into the mechanisms of plasma membrane protection during hyperosmotic stress.

The study presented here, in combination with previous reports [4, 24, 41, 43, 50, 60–62, 64–76, 97–99], provides a detailed snapshot on the multiple cellular functions affected by HOG signaling (Additional file 11: Table S6). In previous efforts from Trempolec et al. a similar descriptive snapshot [100] has been provided for the MAPK p38, the mammalian homologue of Hog1. While we found some interesting overlaps, such as endocytosis [5, 101], our study relies on a systematic, MS-based approach and could therefore provide a more complete picture on MAPK signaling and its impact on cellular processes.

In summary, generating new strategies that could potentially circumvent the incomprehensiveness and stochasticity of MS shotgun data, is pivotal in the wake of “big data” [102–105]. In this report we could demonstrate that efforts in providing tools for re- or combinatorial analysis could be a powerful way to fully leverage MS datasets. Though clearly these efforts should further encompass biological validation experiments, as well as an enhanced biological interpretation of the phosphoproteomics data, the choice of software and the combinatorial use of it seem to be a tangible parameter when comparing interlaboratory results.

## Additional files

Additional file 1: **Figure S1.** Related to Fig. 1. (A) Experimental workflow for LC-MS shotgun experiments. SILAC: stable isotope labeling with amino acids in cell culture, MS: mass spectrometry, TiO_2_: titanium dioxide, SCX: strong cation exchange. (B-D) Histograms of MS-signal intensities of precursor ions (B), PSM scores (C), and peptide lengths of MQ-derived (top) and PD-derived (below) datasets. Light blue bins indicate the distribution of spectra identified solely by MQ. Yellow bins: PD. Green bins: overlap. *P*-values were calculated using the Mann-Whitney *U*-test. (E) Correlation of SILAC log_2_-ratios of mutually quantified phosphorylation sites of setup I + 5′S. (F) Histogram illustrating distribution of SILAC-ratio quantification difference (calculated as MQ/PD SILAC-ratio [log_2_]) of mutually quantified phosphorylation sites of setup I + 5′S. Lines indicate limits of +/− 1 quantification difference. (G) and (H) Results obtained for setup I + 10′S are illustrated similarly to (E) and (F). (I) and (J) Setup I + 0′S. (K) Venn diagrams showing percentage and total number of peptide identifications (IDs) obtained from a MS test run of a HeLa cell extract sample (left) and the dataset described in [4]. Light blue: MQ, yellow: PD, green: overlap. (PDF 21695 kb)

Additional file 2: **Figure S2.** Related to Fig. 3. (A) Heatmap showing SILAC ratios of Hog1-dependent (Romanov et al., 2017 [4] and MQ-derived) phosphopeptides at 0, 5, 15 and 30 min after treatment with 0.5 M NaCl of a wild type (left) or *hog1*Δ (right) strain. (B) Similar to (A) except that results of Hog1-independent phosphorylation sites are shown. (C) Stratification of Hog1-dependent (first two pies) and -independent (last two pies) sites into clusters as defined by Kanshin et al., 2015 [22]. (PDF 371 kb)

Additional file 3: **Figure S3.** Related to Fig. 3. (A) Gel mobility shift assays performed on newly MQ-identified putative Hog1-targets coupled to HKMT-myc (Kic1, Vps53, Orm2) upon 0, 5, 30 and 45 min of osmostress (+ 0.5 M NaCl). Arrows indicate bands with altered gel mobility. (B) Gel mobility shift assay of Orm2-HA exposed to 0, 5, 30 and 45 min of elevated salt levels. (C) Gel mobility shift assay of Orm2-HA in an inhibitor-susceptible Hog1as strain treated with SPP86 inhibitor or DMSO (mock) upon elevated salt levels. (D) MS/MS spectrum indicative for Vps53 Ser^790^ phosphorylation. (E) and (F) Illustration of PRM-measured phosphorylation patterns for the Hog1-independent phosphorylation sites Ser^9^, Ser^15^, Ser^22^, Ser^29^, Ser^31^, and Thr^36^ of Orm2 (E) and Ser^512^ of Kic1 (F) upon hyperosmotic stress (+ 0.5 M NaCl) and inhibitor treatment (SPP86). The green box plots represent the (mean) normalized intensities for the respective phosphopeptide(s). The yellow box plots illustrate the normalized intensities for unphosphorylated counter-peptides. Significance was assessed by comparing intensities derived from all pooled inhibitor-treated samples with those from the mock sample (*t*-test). (PDF 1297 kb)

Additional file 4: **Figure S4.** Related to Fig. 3. (A) to (C) Annotated MS2 Spectra and transition product peak pattern indicative for Orm2 Thr^18^ (A), Kic1 Thr^625^ (B) and Kic1 Tyr^634^ (C). Note: Transition product peaks of Orm2 Thr^18^ are well separated from peaks of peptide isoforms. For Kic1 Thr^625^ as well as Kic1 Tyr^634^, however, co-elution of respective phosphorylated peptide peaks hampers unambiguous peak assignment and quantification. #: phosphorylated amino acid. Indicative transitions used for quantification are shown in color-code. Precursor m/z is indicated in bold. (PDF 622 kb)

Additional file 5: **Figure S5.** Related to Fig. 3. Scanned Western blot films showing M-track protein protein proximity results for the individual candidates. (A) Signals obtained from hyperosmotically challenged cells are shown (1 M Sorbitol, 40 min). Areas picked for densitometric analysis using ImageJ are boxed in red. Signals that have been suspended from the analysis are indicated with a red “x”. me3: antibody recognizing me3K9H3; HA: 12CA5 antibody (B) Same as (A) except that results obtained from unstressed cells are shown. (PDF 5103 kb)

Additional file 6: **Table S1.** Related to Fig. 1. Summary of quantified phosphorylation sites over MS experiments with MQ and PD search engines. This table is provided in a separate Excel file (Additional file 1: Table S1). List of quantified phosphorylation sites over MS experiments (4 experimental conditions in total), containing details on which sites were quantified in which search engine (PD, MQ), the ratios in the 4 different conditions, field assignment (similar to [4]), the corresponding protein and whether an S/T-P motif has been found to be phosphorylated. The column “assignment_figure2” provides the underlying data for Fig. 2a, hence whether the corresponding phosphorylation site has been commonly quantified in PD and MQ, quantified in MQ only or quantified in PD only. The additional columns provide data on the quantified phosphorylation sites that have been published in [22] (salt stress at time points 0, 55″, 60″, regulatory clusters), [5] (salt stress at 5′) and [62] (salt stress upon Cdc28 inhibition) and integrated with the PD and MQ datasets. (XLSX 1765 kb)

Additional file 7: **Table S2.** Related to Fig. 2. Summary of Gene Ontologies identified for MQ- and PD-output on Hog1-targets. The table is provided in a separate Excel file (Additional file 7: Table S2). List summarizing gene ontology (GO) results for Hog1-dependent phosphorylation sites based on GO Slim mapping. The file is composed of three sheets, for (a) the MQ-derived, (b) the PD-derived and (c) the combined (union of PD- and MQ-derived sites) sites and their enrichment in certain gene ontologies. Each table contains information on the GO-ID, its name and category, proteins identified for a given GO-term, the fold-enrichment and the *p*-value as well as the adjusted *p*-value. Finally, the tables also contain the GO level, giving the hierarchy level of the given ontology. (XLSX 60 kb)

Additional file 8: **Table S3.** Related to Fig. 3 and Additional file 2: Figure S2. List of quantified phosphorylation sites over MS experiments, containing details on which sites were quantified in which search engine (PD, MQ) and the ratios in the different conditions. (XLSX 212 kb)

Additional file 9: **Table S4.** Related to Fig. 3 and Additional file 3: Figure S3 and S4. PRM results of Kic1-HB, Orm2-HB and Vps53-HB. Peak intensities of transition product peaks used for quantification are listed. Peptides used for normalization are indicated in bold. (XLSX 72 kb)

Additional file 10: **Table S5.** Related to Fig. 1. List summarizing results of the MS analysis performed on a HeLa cell extract. The file is composed of two sheets, for the MQ-derived (version 1.5.2.8), and the PD-derived (version 1.4) results. (XLSX 2994 kb)

Additional file 11: **Table S6.** Related to Fig. 2. Comprehensive list of phosphorylation sites of Hog1-substrates identified in our study, as well as previous studies. For each phosphorylation site, the study providing the evidence for Hog1-dependency and Cdc28-dependency is indicated. We also included information on the type of evidence (MS-based, M-track, in vitro kinase assay). (A) Hog1-target proteins with at least one specific phosphorylation site confidently identified as a direct Hog1-substrate (MS- or other evidence). (B) Candidate proteins that show interaction with Hog1, but do not contain distinct sites that have been identified as Hog1-dependent. (XLSX 20 kb)

Additional file 12: **Table S7.** Related to Figs. 1 and 2. This table is showing the strains and plasmids used in this study. (XLSX 31 kb)

Additional file 13: **Table S8.** Key resource table. (XLSX 36 kb)

## Data Availability

The MS proteomics datasets supporting the conclusions of this article are available in the PRIDE partner repository [8] of the ProteomeXchange Consortium with the dataset identifiers PXD004294 to PXD004300 [7] and PXD011935 documenting the MQ results. PRM datasets have been deposited to PanoramaWeb [12] (https://panoramaweb.org/gXvdQ2.url) and PRIDE (dataset identifier PXD013789). Further information and requests for resources and reagents should be directed to and will be fulfilled by the Lead Contact, Wolfgang Reiter (wolfgang.l.reiter@univie.ac.at). Key resources are listed in Additional file 13: Table S8.

## Abbreviations

DDA: Data dependent acquisition
DTT: Dithiothreitol
ER: Endoplasmic reticulum
FDR: False discovery rate
GO: Gene ontology
HB: Poly-histidine, biotinylation sequence
HKMT: Histone lysine methyltransferase
HOG: High-osmolarity, glycerol
HPLC: High performance liquid chromatography
IAA: Iodoacetamide
IMAC: Immobilized metal affinity chromatography
LC-MS: Liquid chromatography mass spectrometry
LTQ-FT: Linear trap quadrupole – Fourier transform
MAPK: Mitogen activated protein kinase
MQ: MaxQuant
MS: Mass spectrometry
PD: Proteome Discoverer
PRM: Parallel reaction monitoring
PSG: Phosphorylation site group
PSM: Peptide-spectrum-match
RAM: Regulation of Ace2 activity and cellular morphogenesis
SCX: Strong cation exchange
SGD: Saccharomyces Genome Database
SILAC: Stable isotope labeling using amino acids in cell culture
StARkin domain: Steroidogenic acute regulatory protein-related lipid transfer domain
SWATH MS: Sequential windowed acquisition of all theoretical fragment ion mass spectra
YPD: Yeast extract, peptone, D-glucose
