# Supplementary figures and images for: Novel interconnections of HOG signaling revealed by combined use of two proteomic software packages
Source: Cell Commun Signal. 2019 Jun 17;17:66. doi: 10.1186/s12964-019-0381-z (PMC6572760; doi:10.1186/s12964-019-0381-z)

A

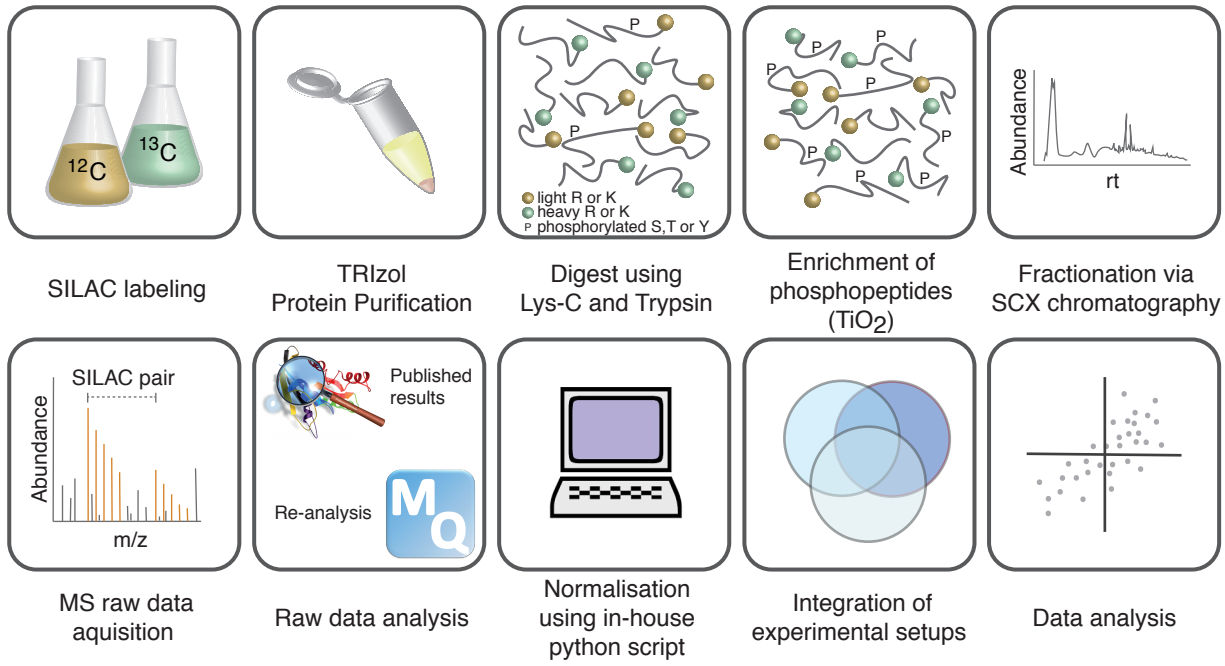

B

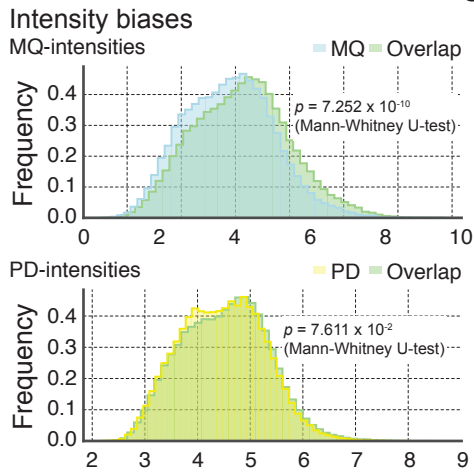

C

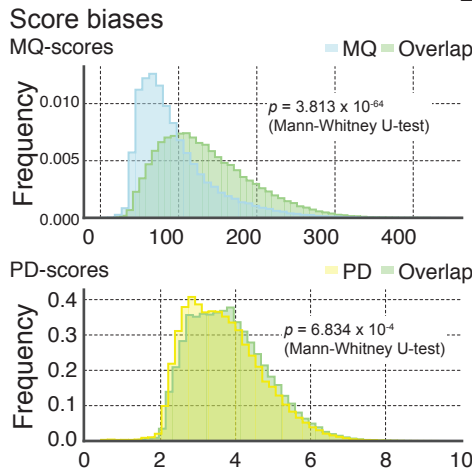

D

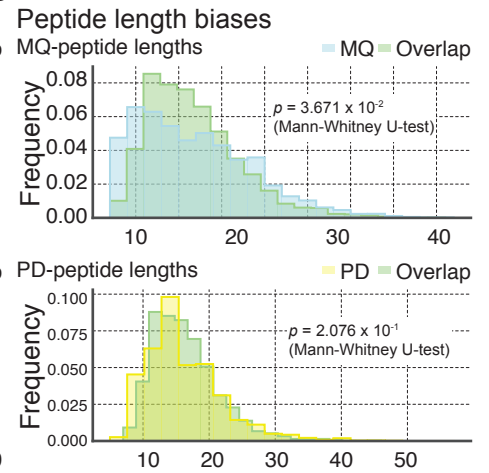

E

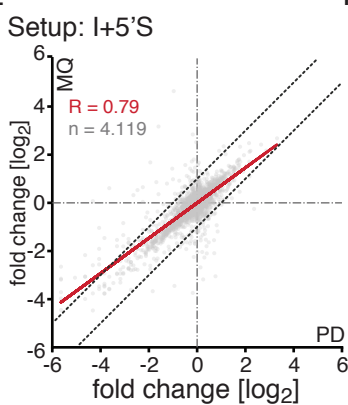

F

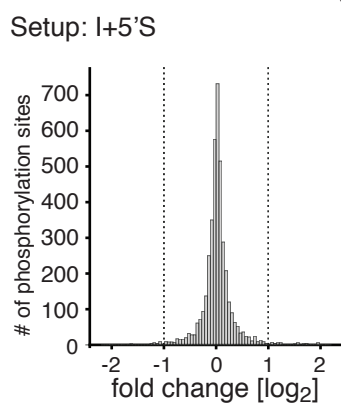

G

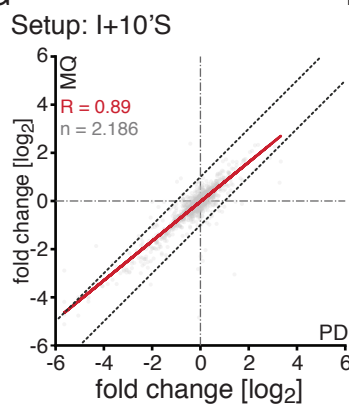

H

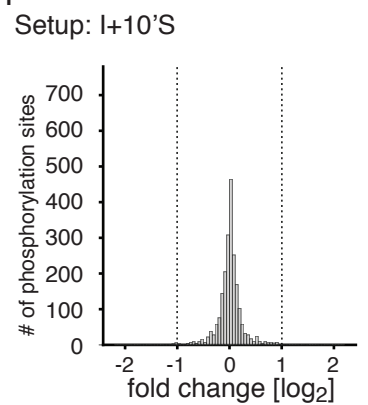

I

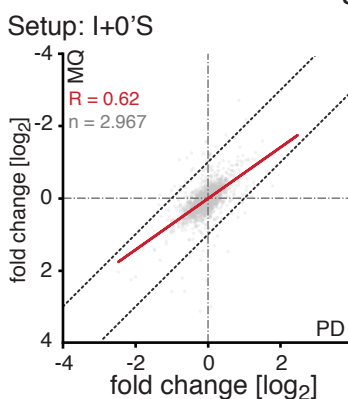

J

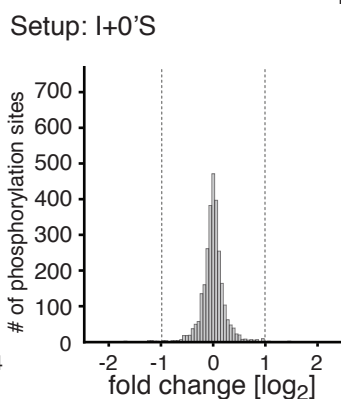

K

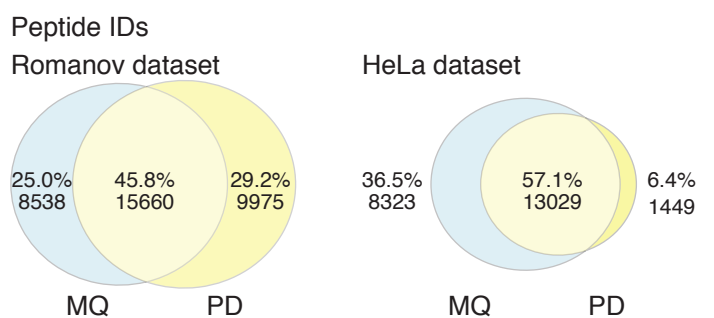

Supplement: Supplementary file 1 — Figure S1. Related to Fig. 1. (A) Experimental workflow for LC-MS shotgun experiments. SILAC: stable isotope labeling with amino acids in cell culture, MS: mass spectrometry, TiO2: titanium dioxide, SCX: strong cation exchange. (B-D) Histograms of MS-signal intensities of precursor ions (B), PSM scores (C), and peptide lengths of MQ-derived (top) and PD-derived (below) datasets. Light blue bins indicate the distribution of spectra identified solely by MQ. Yellow bins: PD. Green bins: overlap. P-values were calculated using the Mann-Whitney U-test. (E) Correlation of SILAC log2-ratios of mutually quantified phosphorylation sites of setup I + 5′S. (F) Histogram illustrating distribution of SILAC-ratio quantification difference (calculated as MQ/PD SILAC-ratio [log2]) of mutually quantified phosphorylation sites of setup I + 5′S. Lines indicate limits of +/− 1 quantification difference. (G) and (H) Results obtained for setup I + 10′S are illustrated similarly to (E) and (F). (I) and (J) Setup I + 0′S. (K) Venn diagrams showing percentage and total number of peptide identifications (IDs) obtained from a MS test run of a HeLa cell extract sample (left) and the dataset described in [4]. Light blue: MQ, yellow: PD, green: overlap. (PDF 21695 kb) [file 12964_2019_381_MOESM1_ESM.pdf]

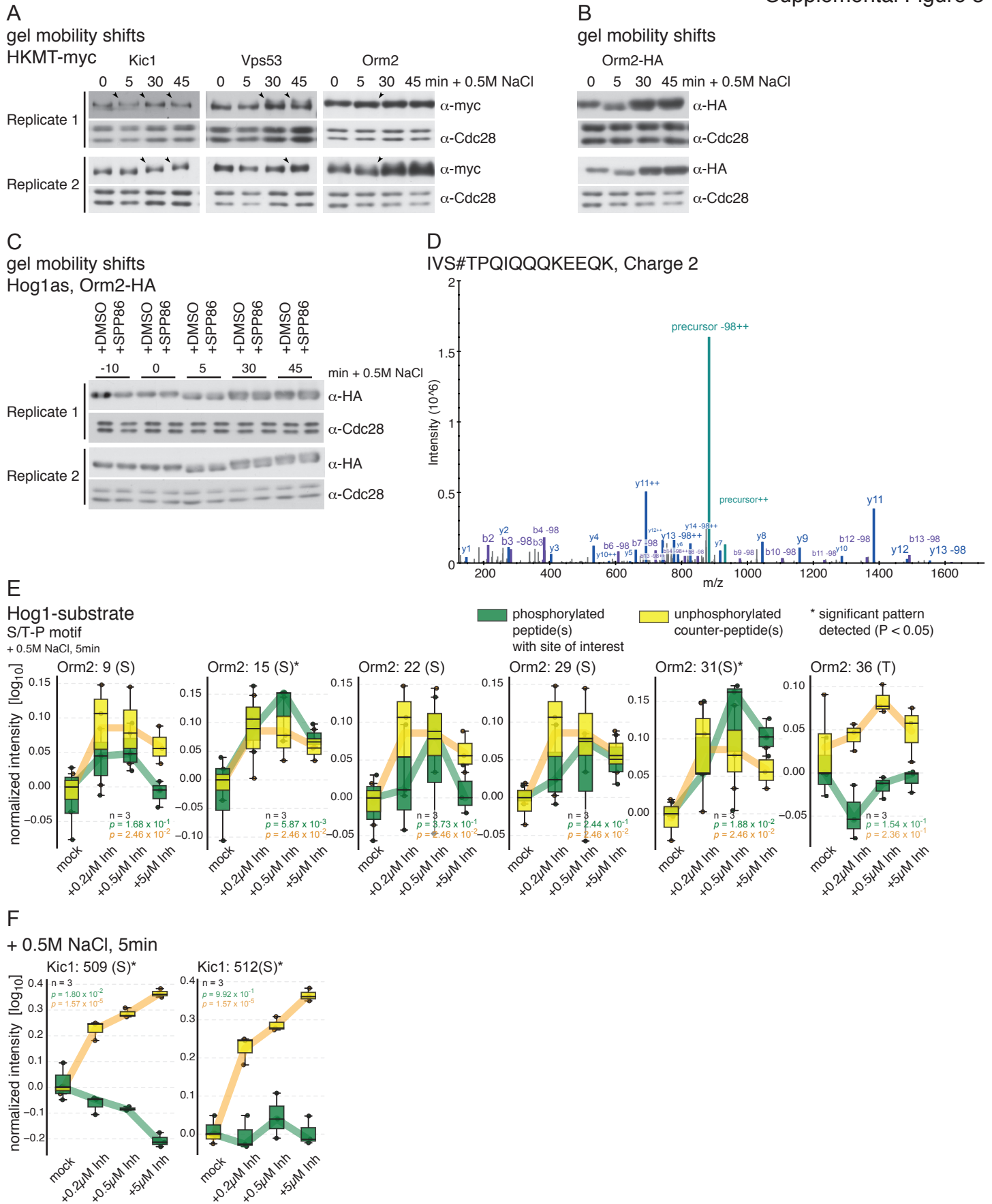

Supplement: Supplementary file 3 — Figure S3. Related to Fig. 3. (A) Gel mobility shift assays performed on newly MQ-identified putative Hog1-targets coupled to HKMT-myc (Kic1, Vps53, Orm2) upon 0, 5, 30 and 45 min of osmostress (+ 0.5 M NaCl). Arrows indicate bands with altered gel mobility. (B) Gel mobility shift assay of Orm2-HA exposed to 0, 5, 30 and 45 min of elevated salt levels. (C) Gel mobility shift assay of Orm2-HA in an inhibitor-susceptible Hog1as strain treated with SPP86 inhibitor or DMSO (mock) upon elevated salt levels. (D) MS/MS spectrum indicative for Vps53 Ser790 phosphorylation. (E) and (F) Illustration of PRM-measured phosphorylation patterns for the Hog1-independent phosphorylation sites Ser9, Ser15, Ser22, Ser29, Ser31, and Thr36 of Orm2 (E) and Ser512 of Kic1 (F) upon hyperosmotic stress (+ 0.5 M NaCl) and inhibitor treatment (SPP86). The green box plots represent the (mean) normalized intensities for the respective phosphopeptide(s). The yellow box plots illustrate the normalized intensities for unphosphorylated counter-peptides. Significance was assessed by comparing intensities derived from all pooled inhibitor-treated samples with those from the mock sample (t-test). (PDF 1297 kb) [file 12964_2019_381_MOESM3_ESM.pdf]

A

## M-track: Western blots

+1M Sorbitol, 40 minutes

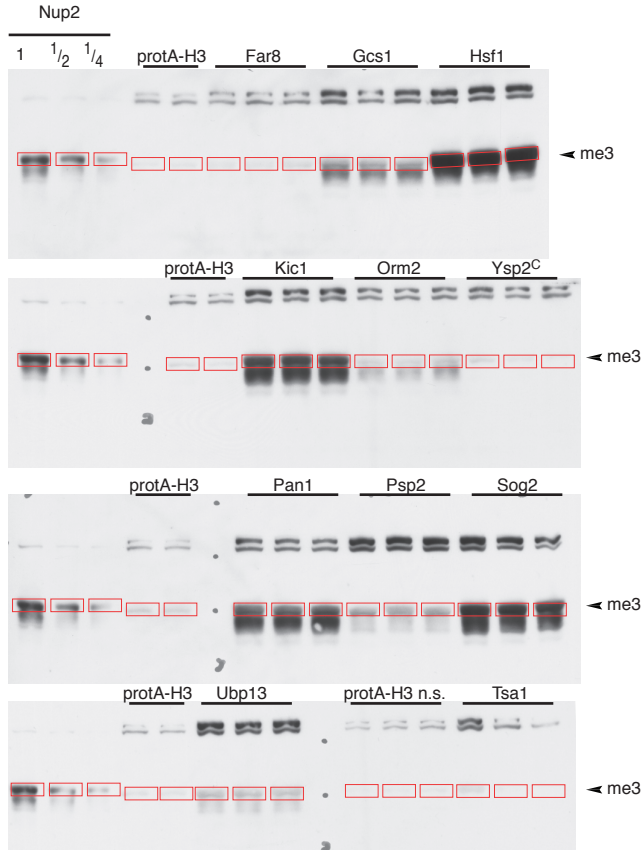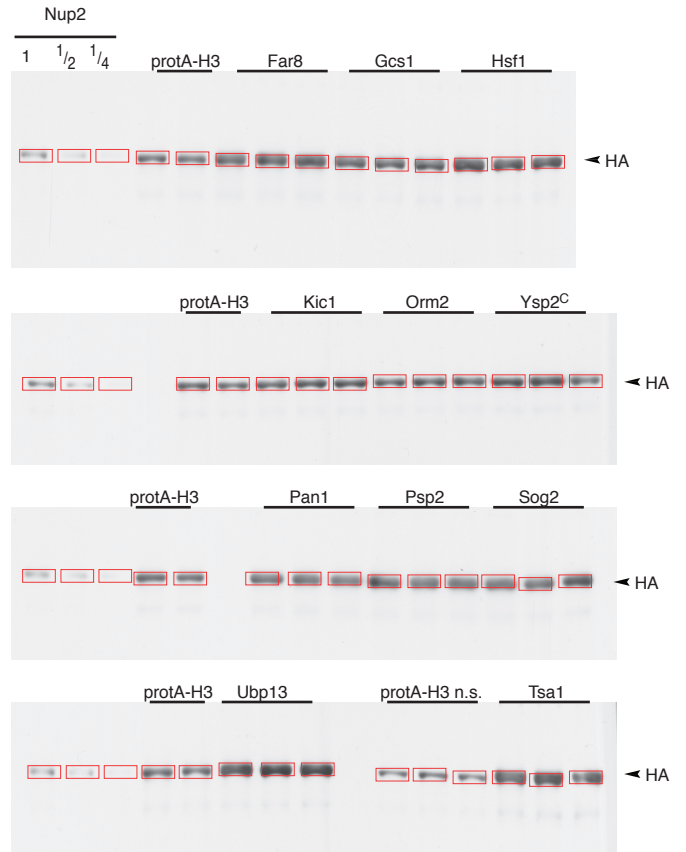

B

unstressed

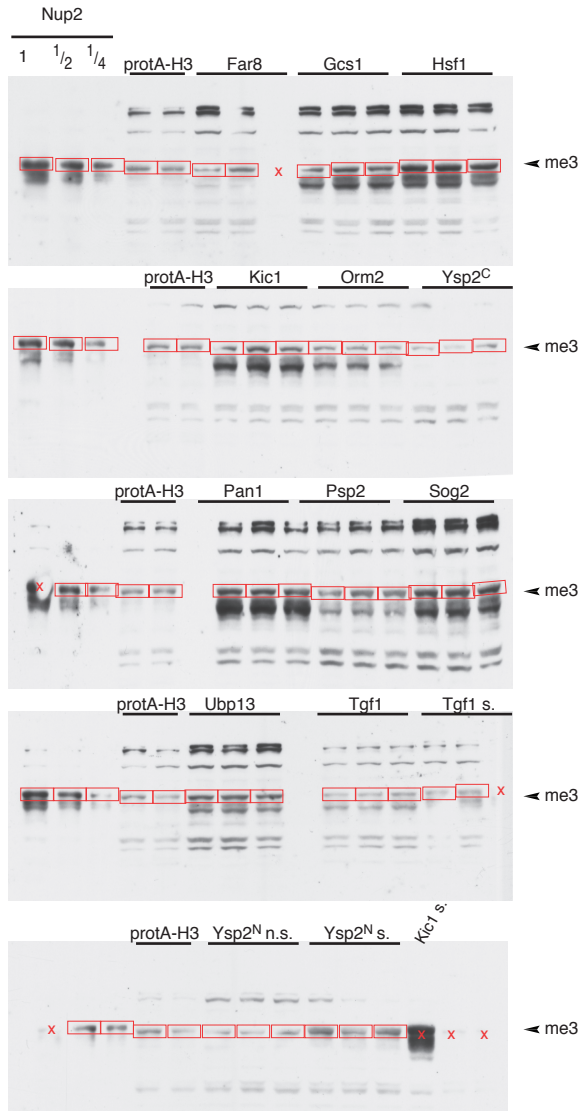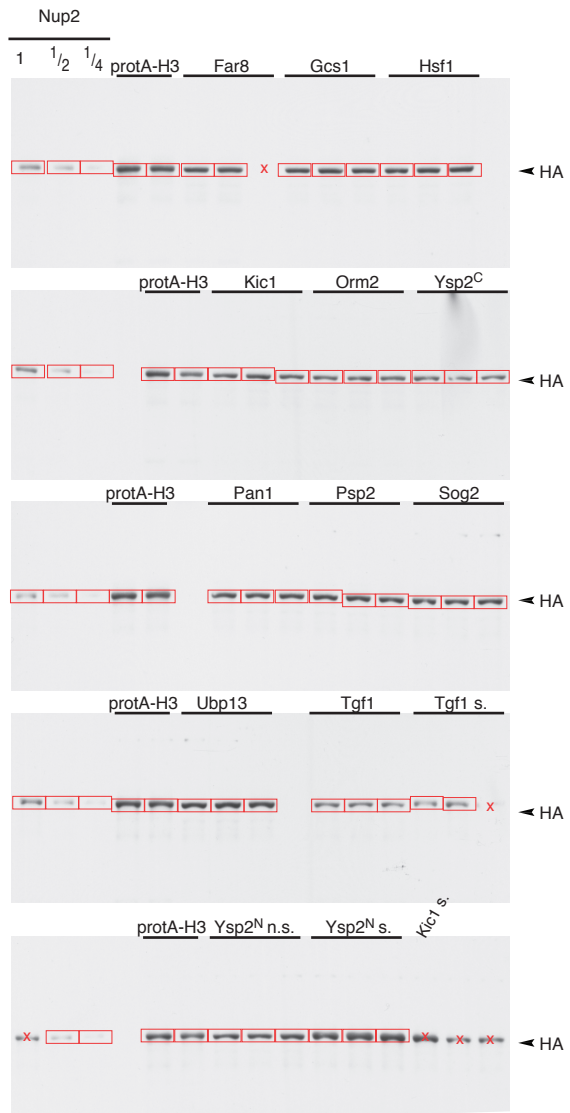

Supplement: Supplementary file 5 — Figure S5. Related to Fig. 3. Scanned Western blot films showing M-track protein protein proximity results for the individual candidates. (A) Signals obtained from hyperosmotically challenged cells are shown (1 M Sorbitol, 40 min). Areas picked for densitometric analysis using ImageJ are boxed in red. Signals that have been suspended from the analysis are indicated with a red “x”. me3: antibody recognizing me3K9H3; HA: 12CA5 antibody (B) Same as (A) except that results obtained from unstressed cells are shown. (PDF 5103 kb) [file 12964_2019_381_MOESM5_ESM.pdf]
