# Supplementary material for: Novel interconnections of HOG signaling revealed by combined use of two proteomic software packages
Source: Cell Commun Signal. 2019 Jun 17;17:66. doi: 10.1186/s12964-019-0381-z (PMC6572760; doi:10.1186/s12964-019-0381-z)

Supplemental Figure 4

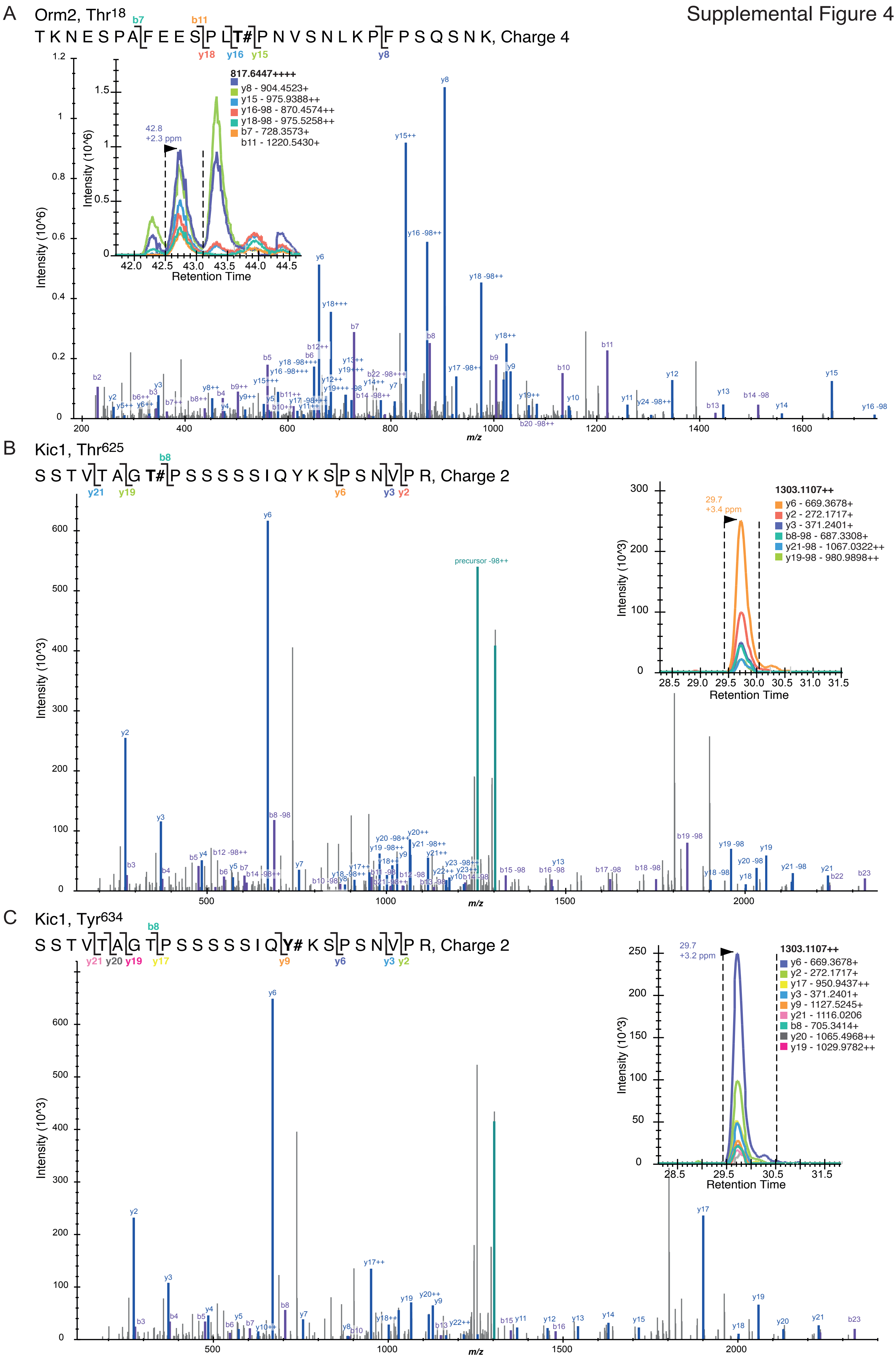

Supplement: Supplementary file 4 — Figure S4. Related to Fig. 3. (A) to (C) Annotated MS2 Spectra and transition product peak pattern indicative for Orm2 Thr18 (A), Kic1 Thr625 (B) and Kic1 Tyr634 (C). Note: Transition product peaks of Orm2 Thr18 are well separated from peaks of peptide isoforms. For Kic1 Thr625 as well as Kic1 Tyr634, however, co-elution of respective phosphorylated peptide peaks hampers unambiguous peak assignment and quantification. #: phosphorylated amino acid. Indicative transitions used for quantification are shown in color-code. Precursor m/z is indicated in bold. (PDF 622 kb) [file 12964_2019_381_MOESM4_ESM.pdf]
