# Supplementary material for: Novel interconnections of HOG signaling revealed by combined use of two proteomic software packages
Source: Cell Commun Signal. 2019 Jun 17;17:66. doi: 10.1186/s12964-019-0381-z (PMC6572760; doi:10.1186/s12964-019-0381-z)

Supplemental Figure 2

A

Hog1-substrates (direct and secondary)

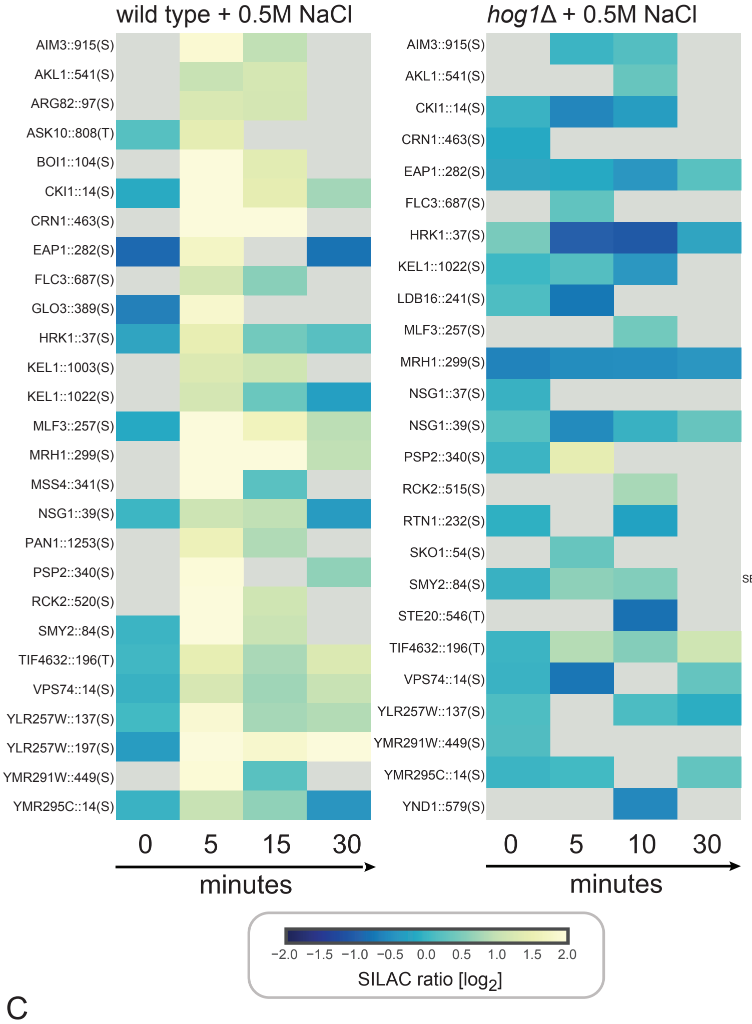

B

Hog1-independent

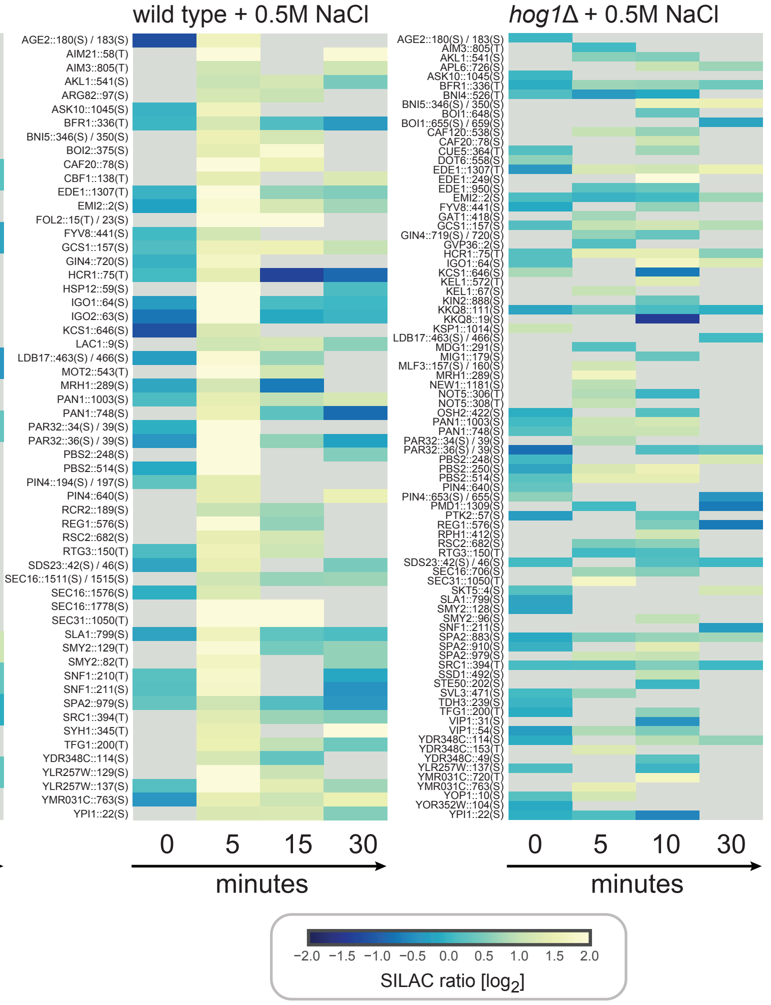

C

Clusteranalysis
acc. to Kanshin et al., 2015

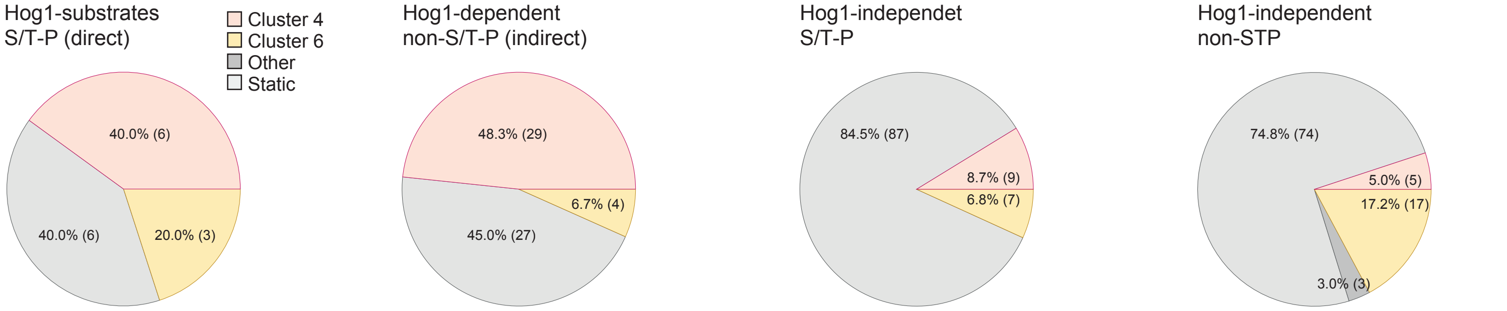

Supplement: Supplementary file 2 — Figure S2. Related to Fig. 3. (A) Heatmap showing SILAC ratios of Hog1-dependent (Romanov et al., 2017 [4] and MQ-derived) phosphopeptides at 0, 5, 15 and 30 min after treatment with 0.5 M NaCl of a wild type (left) or hog1Δ (right) strain. (B) Similar to (A) except that results of Hog1-independent phosphorylation sites are shown. (C) Stratification of Hog1-dependent (first two pies) and -independent (last two pies) sites into clusters as defined by Kanshin et al., 2015 [22]. (PDF 371 kb) [file 12964_2019_381_MOESM2_ESM.pdf]
